# Centrosome and ciliary abnormalities in fetal akinesia deformation sequence human fibroblasts

**DOI:** 10.1038/s41598-020-76192-1

**Published:** 2020-11-09

**Authors:** Ramona Jühlen, Valérie Martinelli, Chiara Vinci, Jeroen Breckpot, Birthe Fahrenkrog

**Affiliations:** 1 Institute of Molecular Biology and Medicine, Université Libre de Bruxelles, 6041 Gosselies, Belgium; 2 Institute of Biochemistry and Molecular Cell Biology, Medical School, RWTH Aachen University, 52074 Aachen, Germany; 3 Center for Human Genetics, University Hospitals Leuven, Catholic University Leuven, Leuven, Belgium; 4 Present Address: Biozentrum, University of Basel, 4056 Basel, Switzerland

**Keywords:** Super-resolution microscopy, Ciliogenesis, Cell proliferation, Nuclear envelope, Developmental biology, Cell biology, Cellular imaging, Cytoskeleton, Mechanisms of disease, Nuclear organization, Post-translational modifications, Molecular biology, Nuclear organization, Post-translational modifications

## Abstract

Ciliopathies are clinical disorders of the primary cilium with widely recognised phenotypic and genetic heterogeneity. Here, we found impaired ciliogenesis in fibroblasts derived from individuals with fetal akinesia deformation sequence (FADS), a broad spectrum of neuromuscular disorders arising from compromised foetal movement. We show that cells derived from FADS individuals have shorter and less primary cilia (PC), in association with alterations in post-translational modifications in α-tubulin. Similarly, siRNA-mediated depletion of two known FADS proteins, the scaffold protein rapsyn and the nucleoporin NUP88, resulted in defective PC formation. Consistent with a role in ciliogenesis, rapsyn and NUP88 localised to centrosomes and PC. Furthermore, proximity-ligation assays confirm the respective vicinity of rapsyn and NUP88 to γ-tubulin. Proximity-ligation assays moreover show that rapsyn and NUP88 are adjacent to each other and that the rapsyn-NUP88 interface is perturbed in the examined FADS cells. We suggest that the perturbed rapsyn-NUP88 interface leads to defects in PC formation and that defective ciliogenesis contributes to the pleiotropic defects seen in FADS.

## Introduction

Fetal akinesia deformations sequence (FADS), the severest form of congenital myasthenic syndromes (CMS), encompasses a broad spectrum of disorders, sharing the inability of the foetus to initiate movement^1,2^. As a result, affected foetuses suffer from multiple joint contractures, including rocker-bottom feet, facial anomalies and lung hypoplasia. In many cases, FADS individuals are born prematurely or stillborn, and a high percentage of live-births die due to respiratory failure^2^.

FADS and CMS can be caused by gene mutations that lead to dysfunction of the neuromuscular system, whereby in 68% of cases the defect is postsynaptic, i.e. in the skeletal muscle cells^3^. The most frequent mutations are found in three genes, namely *RAPSN* (receptor-associated protein of the synapse, rapsyn)^4,5^, *DOK7* (downstream of tyrosine kinase 7)^6^, and *MUSK* (muscle specific kinase)^7,8^, all of which are important regulators of acetylcholine receptor (AChR) formation and maintenance at the neuromuscular junction (NMJ)^9^. The NMJ (also called neuromuscular synapse) is a type of synapse formed between motoneurons and the skeletal muscle fibres that, in vertebrates, use acetylcholine as neurotransmitter^10^. Mutations in the subunits of the muscular nicotinic AChR have also been described in CMS and FADS^9^. We have recently expanded the spectrum of genetic causes for FADS by reporting bi-allelic, loss-of-function mutations in the nucleoporin *NUP88* as cause for a lethal form of FADS^11^.

MuSK and rapsyn are scaffold proteins that play key roles in AChR clustering and NMJ formation. MuSK, a muscle-specific receptor tyrosine kinase, is activated by the extracellular matrix protein agrin upon agrin-binding to Lpr4, a member of the low-density lipoprotein receptor family^12–17^. Activated MuSK induces co-clustering of rapsyn and AChRs^18–21^ and downstream signalling of MuSK requires binding of DOK7 to the phosphotyrosine-binding site in MuSK^22^. Signalling downstream of MuSK is only poorly understood, but it involves interactions of both MuSK and rapsyn to all three cytoskeleton networks, i.e. microtubules, the actin cytoskeleton and intermediate filaments^12,21,23–30^. Rapsyn has been found also in non-muscle cell types^31^ and plays a role in lysosome clustering^23^.

Primary cilia (PC) are discrete, non-motile microtubule-based organelles that protrude from the surface of most quiescent vertebrate cells. PC communicate extracellular signals to the cell, thereby controlling important developmental signalling pathways, such as hedgehog, Wnt, and Notch^32,33^. The core structure of the PC, the axoneme, consists of a microtubule cytoskeleton, comprising a ring of nine doublets that nucleate from the basal body and are surrounded by a ciliary membrane. Doublets consist of a complete A-tubule scaffolding a partial B-tubule^34^. Within the PC, tubulin is subjected to distinct post-translational modifications, in particular acetylation, glutamylation/detyrosination, and glycylation, which control PC assembly and length^35,36^. Additionally, the PC requires a bidirectional transport system, known as intraflagellar transport (IFT), that transfers and correctly localises proteins required for PC growth, maintenance and signalling^34^. The A-tubule binds the IFT retrograde motor protein dynein and the B-tubule binds the IFT anterograde motor protein kinesin-II^34^. Besides IFT, two additional, IFT-independent routes within the lumen of PC are known: passive diffusion and vesicle trafficking^37^. The ciliary proteome is composed of more than 1300 proteins and about 52 subcomplexes^38^. Defects in function or structure of the PC give rise to pleiotropic genetic disorders and manifestations include brain malformations, facial anomalies, neurodevelopmental disorders, such as Joubert syndrome, congenital heart defects, and skeletal malformations^39–41^.

Here, we provide evidence that defects in ciliogenesis contribute to the pleiotropic defects seen in FADS.

## Results

### Nuclei of FADS fibroblasts are frequently misshapen and lobulated

**Figure 1:**
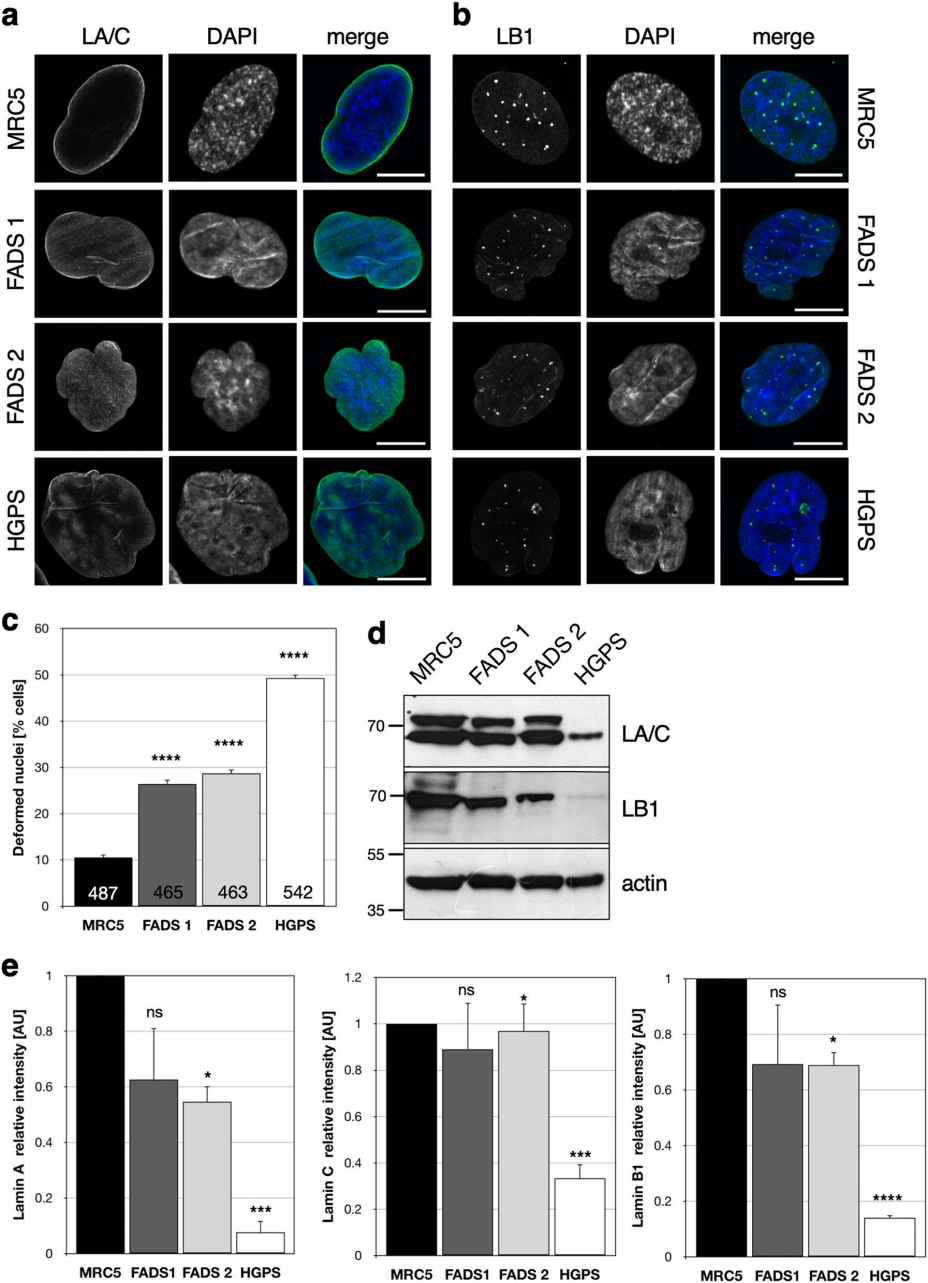
FADS fibroblasts show changes in nuclear morphology. Representative confocal images of fibroblasts from a healthy control foetus (MRC5), two FADS individuals, and a Hutchison Gilford progeria syndrome (HGPS) patient were stained for (**a**) lamin A/C (LA/C, green) and (**b**) lamin B1 (LB1, green), and (**c**) the percentage of deformed nuclei was quantified based on LB1 staining. DNA was visualised with DAPI (blue). Scale bars, 5 µm. Total number of analysed cells is indicated at the bottom of each bar. (**d**) Western blot analysis of LA/C and LB1 expression levels from the indicated fibroblasts and (**e**) their densitometric quantification. Actin was used as loading control. Full-length blots are shown in Fig. S8. ****p < 0.0001, ***p < 0.001, *p < 0.05, ns, *p* not significant, t-test, two-tailed.

While genetic causes and clinical features of FADS are relatively well described, cellular consequences of pathogenic variants in FADS-related genes are largely unknown. We therefore pursued a cell biological characterisation of fibroblasts derived from two FADS individuals: one with unknown genetic cause (FADS 1) and a second one with a homozygous c.484G > A (p.Glu162Lys) variant of *RAPSN* (FADS 2;^5^). Confocal microscopy revealed that fibroblasts derived from these individuals, in contrast to normal human foetal fibroblasts (MRC5), often had nuclei with abnormal shape and a lobulated nuclear envelope (NE), similar to fibroblasts from Hutchinson-Gilford progeria syndrome (HGPS) patients (Fig. 1a,b). About 25–30% of FADS cells exhibited deformed nuclei compared to 50% in case of HGPS, and only 10% of MRC5 cells (Fig. 1c). Nuclear shape and contour irregularities were visualised by immunofluorescence studies with antibodies against lamin A/C (LA/C; Fig. 1a) and lamin B1 (LB1; Fig. 1b). Note the intranuclear lamin B1 puncta in all three fibroblast lines as previously described in other cell lines^42–44^. Despite these similar variations in nuclear morphology in FADS and HGPS fibroblasts, expression of lamin A/C and lamin B1 in FADS fibroblasts was only slightly altered, whereas all three lamin isoforms were significantly reduced in HGPS cells compared to MRC5 cells (Fig. 1d,e).

### FADS fibroblasts are less proliferative

**Figure 2:**
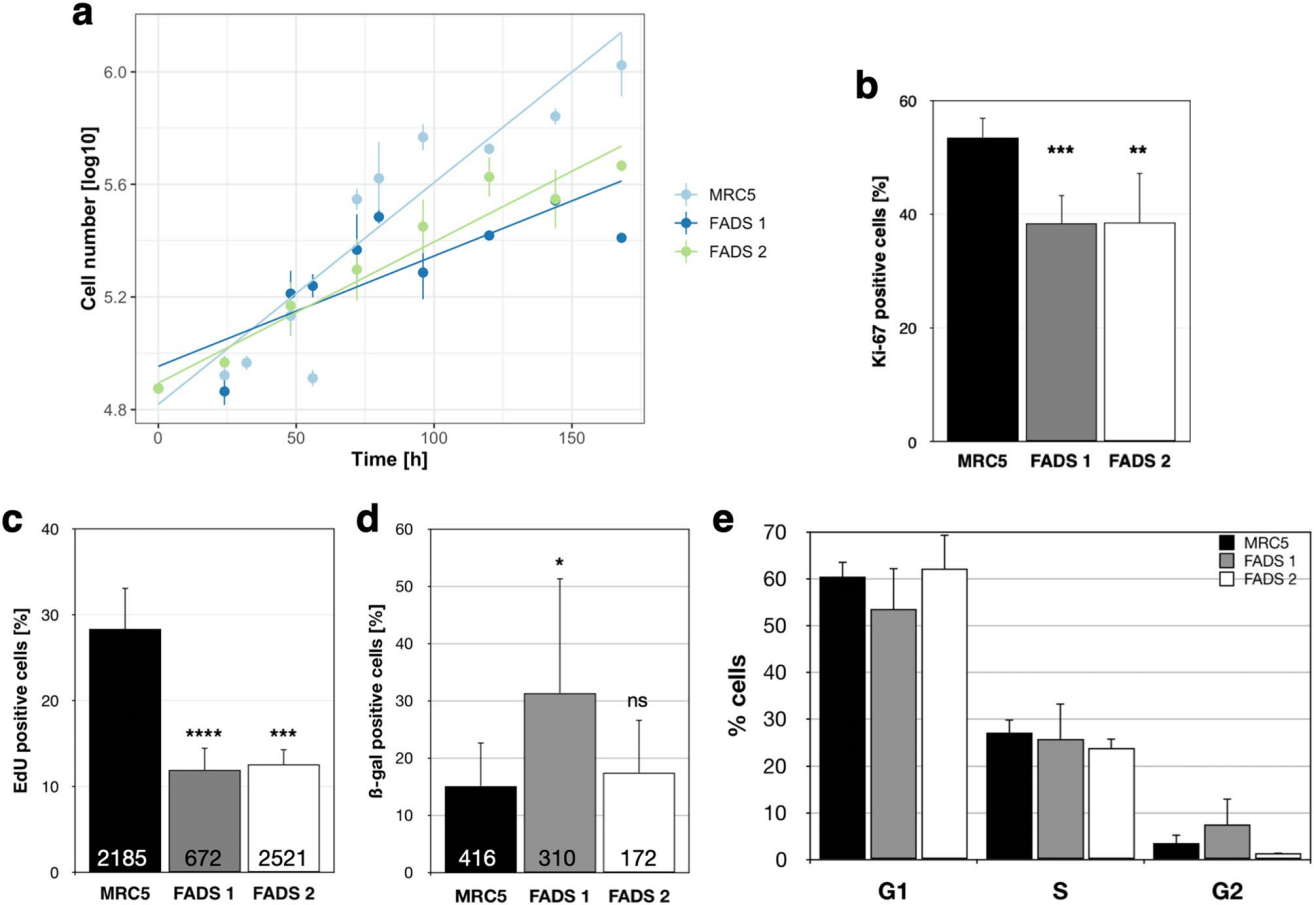
FADS fibroblasts exhibit a decreased proliferative state. (**a**) Equal number of cells were seeded 24 h before measurement and cell numbers were determined at the indicated time points. Data points represent the mean and the point range shows the SEM. p < 0.001, Pearson's χ^2^ test. (**b**) The proliferative state of the indicated fibroblast cell line was determined by immunofluorescence microscopy and anti-Ki-67 staining, (**c)** the proliferative rate by 5-ethynyl-20-deoxyuridine (EdU) incorporation. (**d**) Cellular senescence was evaluated by β-galactosidase staining in FADS and MRC5 fibroblasts. (**e**) Cell cycle distribution of MRC5 and FADS fibroblasts revealed by flow cytometry. Data present the mean ± SD. Total number of analysed cells is indicated at the bottom of each bar. ****p < 0.0001, ***p < 0.001,*p < 0.05, ns, *p* not significant, t-test, two-tailed.

To further characterise FADS fibroblasts in comparison to normal foetal fibroblasts, we subsequently analysed the proliferation of these cells. First, we determined the proliferation rate of cultured FADS fibroblasts in comparison to MRC5 fibroblasts. As shown in Fig. 2a, growth rates of FADS fibroblasts were significantly decreased compared to the MRC5 control cells: the growth constant k, i.e. slope of the linear regression line, was 0.0039 for FADS 1, 0.0050 for FADS 2, and 0.0079 for MRC5. Next, we defined the percentage of cells positive for the proliferation marker Ki-67. Ki-67 marks cells in all active phases of the cell cycle, i.e. G1, S, G2, and M phase^45^. As shown in Fig. 2b, about 55% of MRC5 were in the active phases of the cell cycle, while less than 40% of FADS 1 and FADS 2 cells were Ki-67 positive. To more specifically determine the proliferation rate, we analysed DNA synthesis by incorporation of 5-ethynyl-20-deoxyuridine (EdU), a thymidine analogue which is incorporated into newly synthesised DNA upon replication^46^. EdU incorporation revealed that DNA synthesis in both FADS fibroblast lines was significantly reduced as compared to MRC5 cells (Fig. 2c). The reduced proliferation rate did coincide with a marked increase in cellular senescence in FADS 1, but not FADS 2, fibroblasts, as revealed by β-galactosidase staining (Fig. 2d). Flow cytometry further revealed that the overall cell cycle distribution was similar in FADS and control fibroblasts (Fig. 2e). Together these data indicate that FADS fibroblasts are less proliferative, while their overall cell cycle distribution is not necessarily affected.

### Centrosomal and microtubule nucleation anomalies in FADS fibroblasts

**Figure 3:**
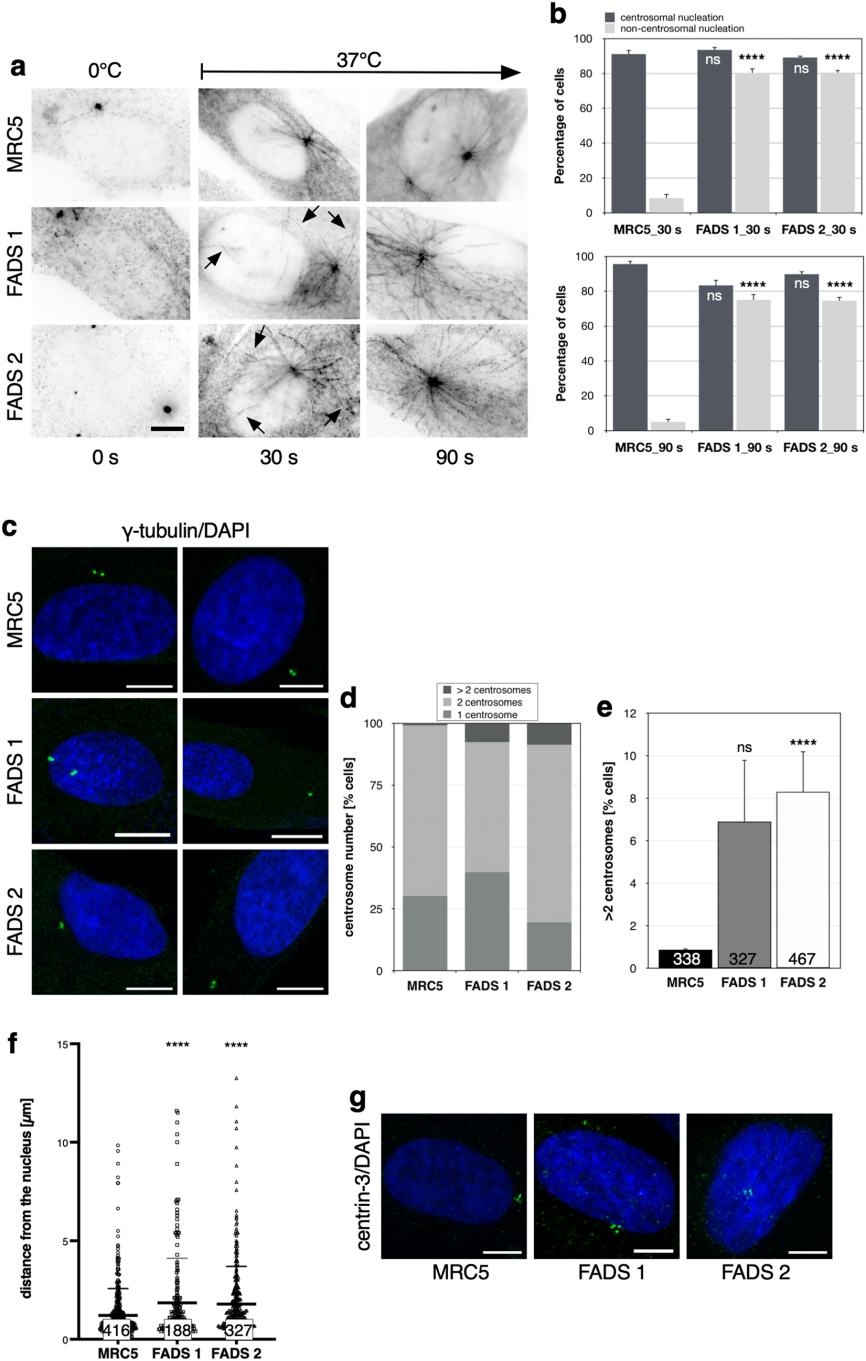
FADS fibroblasts exhibit centrosome abnormalities. (**a**) MRC5 and FADS fibroblasts were subjected to a MT regrowth assay, fixed at the indicated time points, and stained with antibodies against ß-tubulin. Shown are representative inverted immunofluorescence images. Black arrows indicate sites of non-centrosomal MT nucleation at the 30 s time point. (**b**) Quantification of MT nucleation events in the MT regrowth assays. (**c**) Abnormal centrosome number and distance in FADS 1 and FADS 2 fibroblasts as compared to MRC5 cells. Confocal images of cells stained with antibodies against γ-tubulin (green). DNA was stained with DAPI (blue). Quantitative analysis of centrosome number represented as (**d**) stacked bar plots and (**e**) bar plots, and (**f**) their distance to the nucleus represented as scatter plots in the indicated fibroblast cell lines. Data present the mean ± SD. The total number of analysed cells were as indicated. ****p < 0.0001, ns, *p* not significant, t-test, two-tailed. (**g**) Visualisation of centrioles in FADS and MRC5 fibroblasts by anti-centrin-3 antibodies (green) and confocal microscopy. DNA was stained with DAPI (blue). Scale bars, (**a**,**c**) 10 µm, (**g**) 5 µm.

In order to better understand proliferative defects that coincide with FADS and given the link of rapsyn (and MuSK) to the cytoskeleton, we next examined properties of microtubules (MTs) and mitotic spindles in FADS cells. After cold-treatment, we observed abnormal sloppy tubulin spindles in early (prometaphase) and late (telophase) stages of mitotic FADS 1 fibroblasts (Supplemental Fig. S1a), whereas the overall stability of MTs using high and low concentrations of nocodazole was not altered in FADS 1 fibroblasts: in both cell types all MTs disappeared after nocodazole treatment (Supplemental Fig. S1b). When monitoring the regrowth of MTs after cold-induced depolymerisation, regrowth from the centrosome near the NE was observed within 30 s of rewarming to 37 °C in MRC5 cells (Fig. 3a, 30 s). In FADS 1 and FADS 2 cells, however, MT regrowth was initiated at random sites in the cytoplasm during the first 30 s of rewarming. After 90 s both FADS 1 and FADS 2 fibroblasts showed increased MT polymerisation/growth speed compared to the control (Fig. 3a, 90 s). Non-centrosomal (nc) MT polymerisation was observed in less than 10% of MRC5 cells, whereas 70–80% of FADS 1 and FADS 2 showed both, centrosomal and ncMT regrowth (Fig. 3b, 30 s and 90 s).

The centrosome is generally the main microtubule organizing centre (MTOC) and responsible for nucleating MTs^47^. Due to the ncMT nucleation observed in FADS fibroblasts, we next analysed their centrosome number and position. Staining of fibroblasts with γ-tubulin antibodies (Fig. 3c) revealed that FADS fibroblasts have abnormal centrosome numbers, i.e. more than two (Fig. 3d,e). Whereas only about 1% of MRC5 cells had more than two centrosomes, in FADS fibroblasts the percentage increased to 7–8%. Abnormal centrosome number in FADS 1 and FADS 2 did not coincide with an alteration in cell cycle distribution (Fig. 2e). Moreover, centrosomes in FADS cells were more frequently found at larger distance from the nucleus in comparison to control cells (Fig. 3f). Despite the abnormal centrosome number in FADS fibroblasts, immunostaining with centrin-3 antibodies revealed that each centrosome is comprised of two centrioles (Fig. 3g), indicating that the centrioles duplicate normal.

### Ciliary abnormalities in FADS fibroblasts

**Figure 4:**
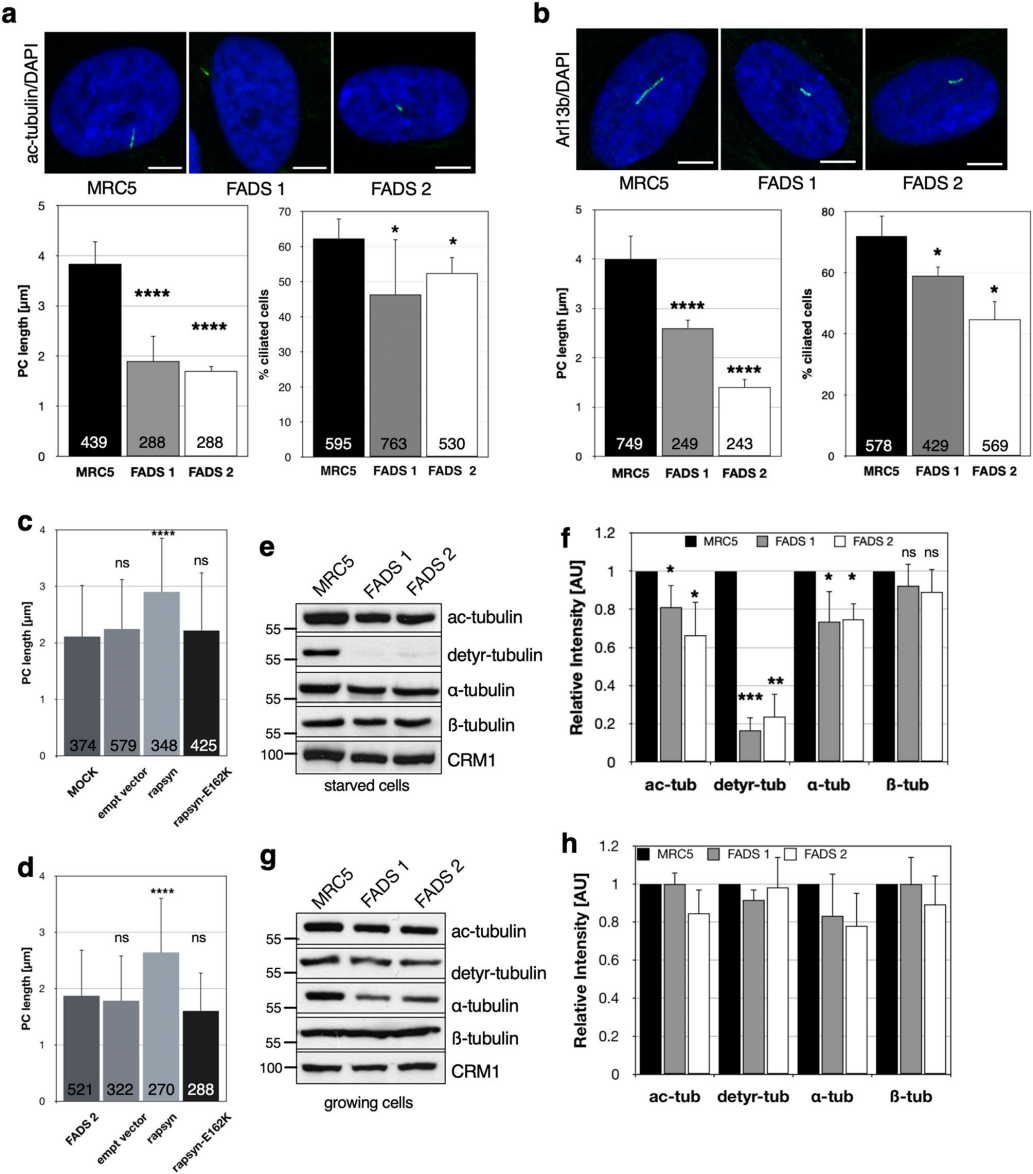
Ciliogenesis is impaired in FADS fibroblasts. Representative confocal images of (**a**) anti-acetylated α-tubulin (ac-tubulin, green) and (**b**) anti-Arl13b (green) immunofluorescence staining in MRC5, FADS1, and FADS2 fibroblasts alongside with quantification of primary cilia (PC) length and percentage of ciliated cells are shown. Fibroblasts were subjected to serum-starvation 48 h before fixation and staining. Expression of Venus1-tagged rapsyn (**c**) or FLAG-tagged rapsyn (**d**) led to a rescue of PC length in FADS 2. Data present the mean ± SD. Lysates from (**e**) serum-starved and (**g**) growing fibroblasts were subjected to Western blot analysis using antibodies against ac-tubulin, detyrosinated (detyr) α-tubulin, α-tubulin, and ß-tubulin. CRM1 was used as loading control. The respective full-length blots are shown in Fig. S9 (starved) and S10 (growing). Relative intensity of ac-tubulin, detyr-tubulin, α-tubulin, ß-tubulin levels in (**f**) serum-starved and (**h**) growing fibroblasts as determined by densitometry. Total number of analysed cells is indicated at the bottom of each bar. Number of experiments for ac-tubulin staining were: n = 6, MRC5, FADS 1; n = 3, FADS 2. Number of experiments for Arl13b staining were: n = 6, MRC5; n = 3, FADS 1, FADS 2. ****p < 0.0001, ***p < 0.001, **p < 0.01, *p < 0.05, ns, *p* not significant, t-test, two-tailed. Scale bars, 10 µm.

Centrosome abnormalities are frequently associated with human disease, not only cancer, but also ciliopathies^48^. Considering the pleiotropic developmental abnormalities that characterises fetal akinesia, we explored whether defects in primary cilia (PC) formation may contribute to the disease. To address this question, MRC5 control and FADS fibroblasts were subjected to serum-starvation and PC formation was analysed by immunofluorescence microscopy using antibodies against acetylated α-tubulin and Arl13b (ADP ribosylation factor like GTPase 13b), a ciliary marker protein. By both, α-tubulin (Fig. 4a) and Arl13b (Fig. 4b) immunostaining, we found that PC were significantly shorter in FADS fibroblasts compared to control cells, and the percentage of ciliated cells was smaller (Fig. 4a,b). Similarly, we observed PC defects in FADS fibroblasts grown on crossbow-shaped micropatterns and stained for acetylated and detyrosinated α-tubulin (Supplemental Fig. S2a).The reduced PC length appears to arise from defects in cilia growth, as PC resorption in FADS fibroblasts was similar to control cells, i.e. not enhanced (Supplemental Fig. S2b). In contrast to FADS, HGPS and other laminopathies do not coincide with PC defects (Supplemental Fig. S2c). Expression of wild-type Venus 1-tagged rapsyn (Fig. 4c) or FLAG-tagged rapsyn (Fig. 4d) in FADS 2 cells, which harbour the rapsyn E162K mutant protein, rescued the ciliary length defect in these cells, in contrast to mutant E162K rapsyn and the respective empty vectors, confirming that the FADS-causing mutation in *RAPSN* contributes to the ciliary defects. Additionally, the cellular distribution of detyrosinated α-tubulin in FADS cells appeared more aggregated in immunofluorescence staining (Supplemental Fig. S2d).

PC growth and length is regulated by changes in post-translational modifications (PTMs) of α-tubulin, in particular acetylation, detyrosination, and glycylation of α- and β-tubulin^35,49^. The levels of detyrosination increase during PC assembly^49^. By immunoblotting, we found significantly lower levels of detyrosinated α-tubulin in serum-starved FADS fibroblasts (Fig. 4e,f), consistent with the observed assembly defects.

Also, acetylated α-tubulin and total α-tubulin were markedly reduced, while the levels of β-tubulin were comparable in FADS and MRC5 control cells (Fig. 4e,f). Variations in α-tubulin PTMs and overall tubulin levels in growing (i.e. non-starved) cells were not significant (Fig. 4g,h). Due to a lack of appropriate antibodies against glycylated tubulin, we could not investigate glycylation of α- and β-tubulin in our fibroblasts.

### Depletion of FADS proteins causes ciliary defects

**Figure 5:**
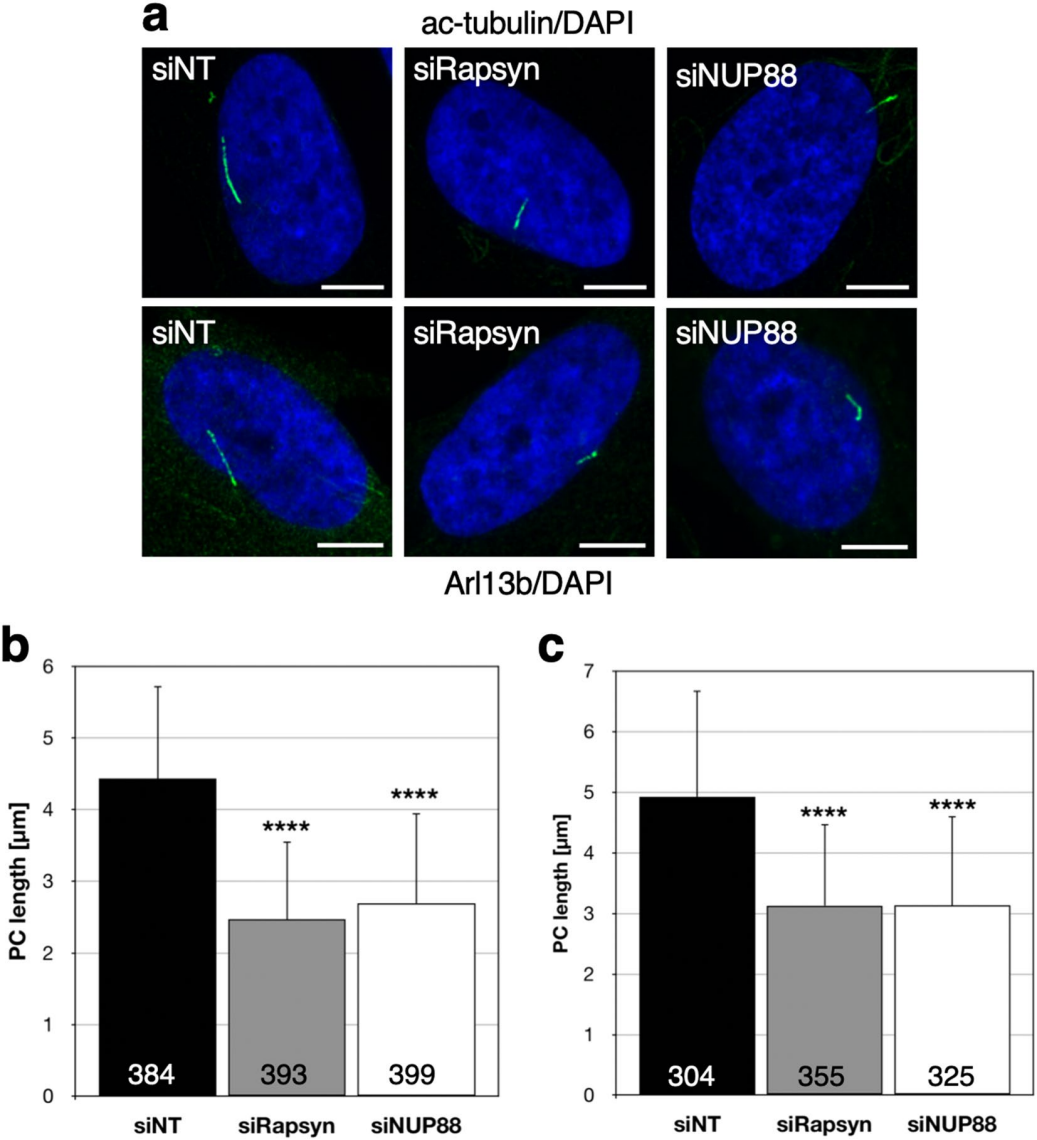
FADS proteins are critical for ciliogenesis. (**a**) Effects of control (siNT), rapsyn (siRapsyn) or NUP88 (siNUP88) depletion in MRC5 cells subjected to serum starvation and ciliogenesis. Representative confocal images of cilia marker acetylated α-tubulin (ac-tub; green) and Arl13b (green), and DNA (blue) are shown. Quantification of primary cilia (PC) length are shown for (**b)** acetylated α-tubulin and (**c)** Arl13b. Data present the mean ± SD. Total number of analysed cells were as indicated. ****p < 0.0001, t-test, two-tailed. Scale bars, 10 µm.

To support the idea that FADS in more general is a ciliopathic disease, we next depleted rapsyn or NUP88 from MRC5 fibroblasts by RNA interference. Mutations in either *RAPSN* or *NUP88* are known genetic causes for FADS^4,5,9,11^. Forty-eight hours after siRNA transfection, cells were subjected to serum-starvation to initiate cell cycle exit and PC formation. We found that PCs were significantly shorter upon the respective depletion of rapsyn and NUP88 from MRC5 cells, as revealed by acetylated α-tubulin (Fig. 5a,b) and Arl13b (Fig. 5a,c) staining and immunofluorescence microscopy. Similarly, siRNA-mediated depletion of MuSK, another key factor in the pathogenesis of FADS, resulted in shorter PC (Supplemental Fig. S3a). Depletion levels of rapsyn and NUP88 were determined by Western blot and bands quantified by densitometry (Supplemental Fig. S3b-d).

### FADS proteins localise to centrosomes and cilia

**Figure 6:**
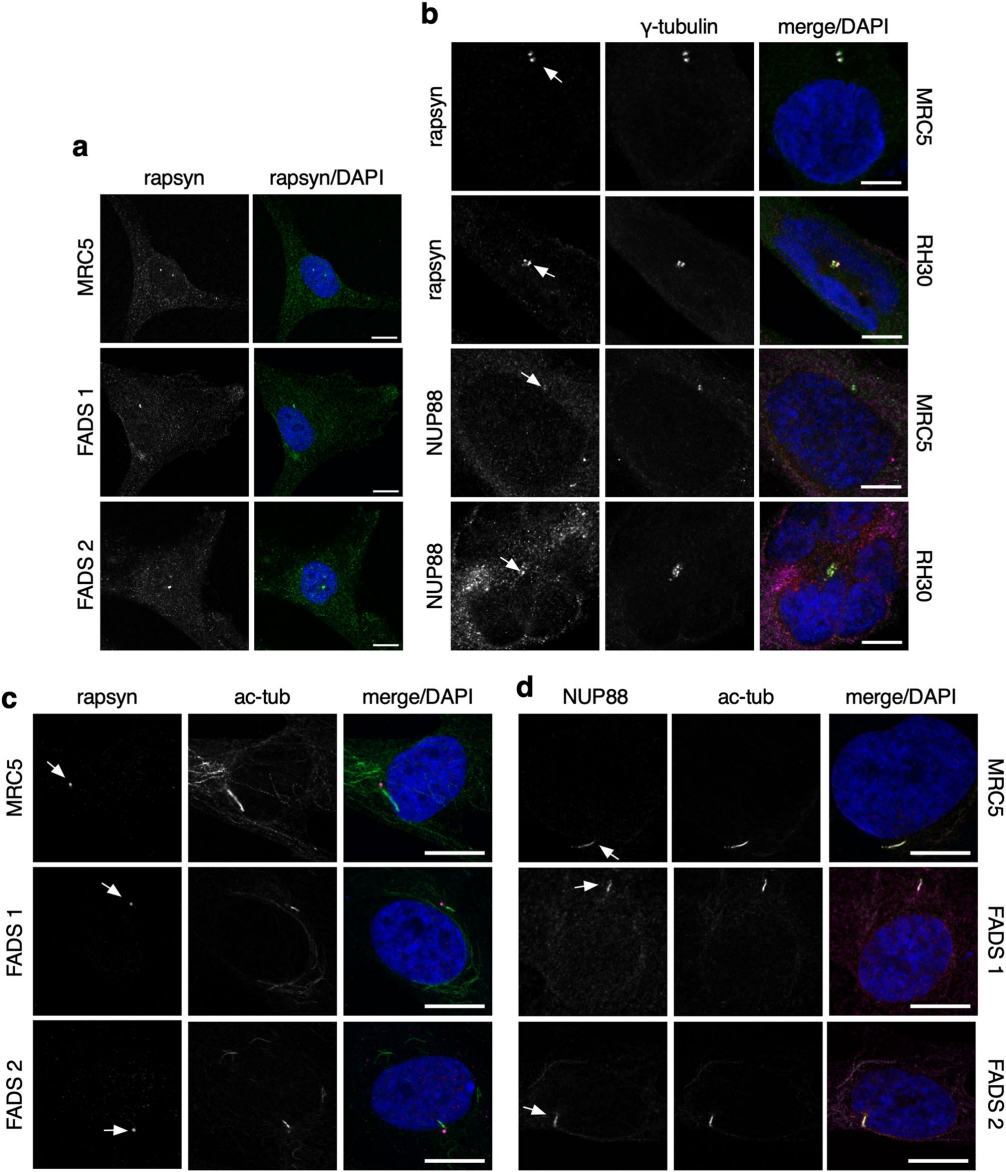
Localisation of rapsyn and NUP88 at centrosomes and primary cilia. (**a**) MRC5 and FADS cells were stained for rapsyn (green) to reveal its intracellular localisation in fibroblasts. (**b**) Images of MRC5 and RH30 cells stained for rapsyn (magenta) or NUP88 (magenta), co-stained for γ-tubulin (green). (**c**) Rapsyn (magenta) and (**d**) NUP88 (magenta) localise to the acetylated α-tubulin (ac-tub) stained green primary cilium (green) in serum-starved MRC5, FADS1, and FADS 2 fibroblasts. DNA stained by DAPI (blue). Shown are representative confocal images. Arrows indicate the distinct localisation of the proteins at primary cilia. Scale bars, 10 µm.

We next determined the intracellular localisation of rapsyn in MRC5 and FADS fibroblasts. As shown in Fig. 6a, rapsyn is distributed throughout the cytoplasm and enriched at centrosomes in all three cell lines. To confirm the association of rapsyn with centrosomes, we performed co-localisation experiments and found that rapsyn indeed co-localised with γ-tubulin at centrosomes in methanol-fixed MRC5 fibroblasts (Fig. 6b). The association of rapsyn with centrosomes was additionally confirmed in the human skeletal muscle precursor rhabdomyosarcoma cell line RH30 (Fig. 6b). Moreover, also NUP88 localised to centrosomes in MRC5 and RH30 cells (Fig. 6b), in contrast to its partner nucleoporins NUP214 and NUP62 or nucleoporins NUP93 and NUP153 (Supplemental Fig. S4a). siRNA-mediated depletion of rapsyn and NUP88 from MRC5 cells validated the specificity of the particular antibodies and the respective localisation of rapsyn (Supplemental Fig. S4b) and NUP88 (Supplemental Fig. S4c) at centrosomes. The specificity of the antibodies was further validated in MRC5 and HeLa cells by quantification of the mean pixel density values (Supplemental Fig. S5a-d).

In view of the strong link between centrosomes and PC^50,51^, we examined the localisation of rapsyn and NUP88 in serum-starved, ciliated MRC5 and FADS fibroblasts. In MRC5 cells, we detected rapsyn at the cilia base (Fig. 6c) and NUP88 along the axoneme of the PC (Fig. 6d). Recruitment of rapsyn and NUP88 to the PC was not affected in FADS 1 and FADS 2 fibroblasts, despite the homozygous point mutation of *RAPSN* (c.484G > A) in FADS 2 cells (Fig. 6c,d; Supplemental Table S1). Besides rapsyn and NUP88, we also detected the FADS protein DOK7 at axoneme in MRC5 and FADS fibroblasts (Supplemental Fig. S6a), in contrast to MuSK, which did not locate to the cilia base or to axoneme (Supplemental Fig. S6b).

**Figure 7:**
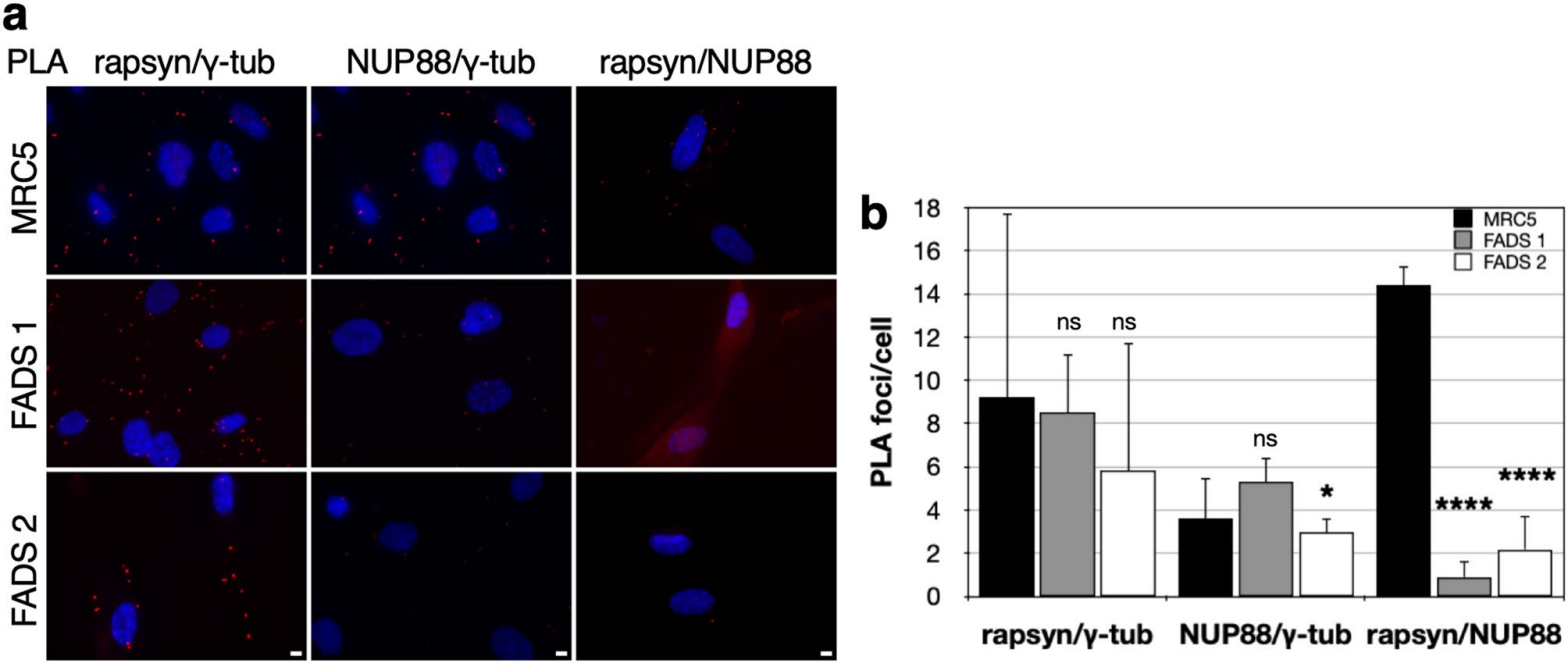
Proximity ligation assays in MRC5, FADS 1 and FADS 2 fibroblasts. (**a**) Close proximity (~ 40 nm) between rapsyn and γ-tubulin (γ-tub), NUP88 and γ-tub, as well as rapsyn and NUP88 was visualised by PLA foci (red) and (**b**) quantified. PLA foci/cell were quantified using Fiji/ImageJ. DNA was stained with DAPI (blue). Shown are representative immunofluorescence images. Scale bars, 10 µm. ****p < 0.0001, *p < 0.05, ns, *p* not significant, t-test, two-tailed.

To further support the link between rapsyn and NUP88 on the one hand and centrosomes and the PC on the other, we next executed proximity ligation assays (PLAs). By PLA, proteins adjacent to the protein of interest with a maximum distance of 40 nm can be detected as PLA amplification foci (Duolink PLA Troubleshooting Guide). As shown in Fig. 7a, PLA foci in the cytoplasm of MRC5 fibroblasts were detected for rapsyn as well as for NUP88 with γ-tubulin. The number of rapsyn/γ-tubulin PLA foci per cell did not vary significantly between MRC5 and FADS fibroblasts (Fig. 7b). In case of NUP88, the number of NUP88/γ-tubulin PLA foci per cell in FADS 2 fibroblasts was similar to MRC5, whereas the number was slightly increased in FADS 1 (Fig. 7b). Additionally, PLA foci in MRC5 were detected for rapsyn and NUP88 (Fig. 7a). The number of rapsyn/NUP88 PLA foci per cell was significantly reduced in FADS 1 and FADS 2 fibroblasts (Fig. 7a,b). Control PLAs are presented in Supplemental Fig. S7a.

## Discussion

FADS is a clinically and genetically heterogeneous constellation, which is characterised by reduced or lack of foetal movement in utero. While in the past few years, due to the increased availability of next generation sequencing, significant advances have been made in understanding the molecular aetiology of FADS, the cellular consequences of FADS-related mutations have previously remained largely unstudied. Here we provide evidence that FADS coincides with defects in ciliogenesis, which may account for the pleiotropic morphological defects seen in FADS individuals. We show that primary cilia (PC) length is reduced in fibroblasts derived from two FADS individuals (Fig. 4) and that the respective depletion of the FADS proteins rapsyn, NUP88 and MuSK from control fibroblasts is similarly resulting in shorter PC (Fig. 5, Supplemental Fig. S3a). Hence, a ciliopathic disease underlies the pathogenesis of FADS, at least in a subset of patients.

### FADS fibroblasts have shorter primary cilia

Important for the regulation of PC length are tubulin post-translational modifications (PTMs). PTMs of α-tubulin have been linked to a variety of human pathogenic phenotypes^52^. Axonemal microtubules (MTs) are a common target for several PTMs, and the amount of PTMs increases during maturation of the cilium, distinguishing mature cilia from assembling/immature cilia^53^. Stable MTs are marked by acetylation, and axonemal MTs are a common target for acetylation contributing to ciliogenesis and ciliary mechano-sensing^54,55^. Detyrosinated α-tubulin is enriched at the B-tubules^56^. Since the anterograde intraflagellar transport (IFT) moves along B-tubules, detyrosination has been suggested to stimulate anterograde IFT^57^. The ‘tubulin code’, which defines, besides α-tubulin PTMs, also the unique composition of the different tubulin isotypes, is a crucial characteristic of axonemal MTs^58^. Indeed, in *C. elegans* it has been shown that the loss of the α-tubulin isotype leads to abnormal axoneme ultrastructure and impaired cilia transport^59^. We hypothesise that a distinct alteration in the ‘tubulin code’, including PTMs, in FADS cells adds to the observed PC defects and probably results in a decreased amount of mature cilia (Fig. 4, Fig. 5). We consider indeed defects in anterograde IFT and PC maturation as causative for the reduced PC length and number in FADS, as resorption of PC is similar in FADS and control fibroblasts (Supplemental Fig. S2b), indicating a regular retrograde IFT. Axonemal MTs can be seen as extensions of the cytoplasmic MT network, which plays an important role in the regulation of cilia by maintaining the balance of available tubulin molecules^60^. We are currently examining how the cytoplasmic MT network is organised in FADS cells and may contribute to PC impairments. It will further be interesting to see whether altered MT network in FADS cells contributes to the decreased proliferation rate accompanied by nuclear shape irregularities we observed in our study. A link between an abnormal microtubule network and cell cycle progression has recently been described in oral-facial-digital type I (OFD1), another ciliopathic disease^61^.

### The role of rapsyn and NUP88 in ciliogenesis

**Figure 8:**
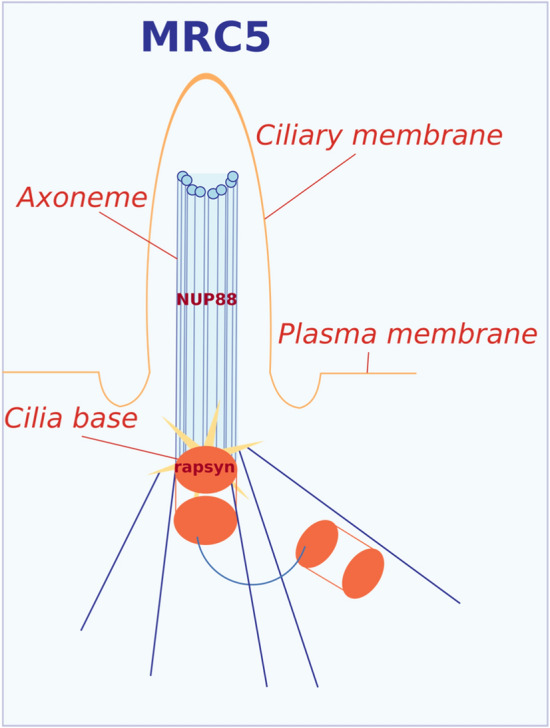
Schematic representations summarising the association of rapsyn and NUP88 with primary cilia in MRC5 fibroblasts.

How do rapsyn and NUP88 come into the game? A striking connection between nucleoporins, the nucleocytoplasmic transport machinery and cilia is known since quite some years, but the function of nucleoporins at cilia has remained largely obscure (for review see:^62^). A proteomic screen has recently identified NUP88 as part of the cilia proteome^38^. Consistent with the localisation of NUP88 to the axoneme of ciliated cells (Figs. 6d, 8), NUP88 appears to be part of the Bardet-Biedl Syndrome (BBSome) coat complex, in complex with DYNLT1, and the WDR19/IFT-A complex^38^. Similar to NUP88, we also detected its complex partners NUP214 and NUP62 at the axoneme, in contrast to other nucleoporins, such as NUP98 and NUP93 (Supplemental Fig. S4b). In contrast to our data presented here, NUP214 and NUP62 have both been previously localised to the cilia base and transition zone^63,64^. This discrepancy might arise from two obvious differences: we examined the endogenous proteins by employing antibodies, whereas in previous studies epitope-tagged versions of NUP214 and NUP62 were analysed. Alternatively, species-specific differences between human (in our study) and rodent (rat or mice in the previous studies) cells/cilia may play a role. Further studies are required to untangle this inconsistency.

NUP88 together with NUP214 and NUP62 has crucial roles in nuclear export mediated by the nuclear export receptor CRM1^65,66^. CRM1 carries cargoes from the nucleus to the cytoplasm in complex with by the small GTPase Ran. Emerging evidence now suggest that CRM1 also regulates non-centrosomal (nc) MT nucleation in a RanGTP-dependent mechanism, and that depletion of CRM1 in yeasts results in increased ncMT nucleation after cold-treatment ^67^. Furthermore, Gle1, which has a crucial role in mRNA export and is closely associated with the nuclear pore complex, has been shown to locate to centrosomes and the basal body, and to regulate MT organisation^68^. It is tempting to speculate that the increased levels of random ncMT nucleation after cold-treatment in FADS cells (Fig. 3a) are caused by similar mechanisms, and thus impair PC formation, alter mitotic spindles (Supplemental Fig. S1a) and concomitantly result in the lower proliferation rates of FADS fibroblasts (Fig. 2). It will be interesting to see how NUP88 is recruited to centrosomes and MTs, and whether this involves similar mechanisms as described for the muscle-specific α-isoform of nuclear envelope protein Nesprin1/SYNE1^69,70^. Of note, mutations in *SYNE1* have also been described in FADS cases^71^.

With respect to NUP88 and its association with the BBSome, it has previously been shown, in *C. elegans* and mice, that defects in BBSome components result in defective IFT, which is important for cilium growth, and morphological defects of the cilia^36,72–75^. This suggests that NUP88 may play a role in IFT as well, and that its loss consequently impairs IFT and cilia growth. Future mechanistic studies should eventually provide more insights in this regard. Interestingly, mutations in WDR19, a binding partner of NUP88 in the BBSome^38^, are associated with, for example, cranioectodermal dysplasia 4 (CED4), a disorder primarily characterised by craniofacial, skeletal and ectodermal abnormalities^76^.

Rapsyn has thus far not been associated with the cilium. The fact that expression of wild-type rapsyn in the FADS 2 fibroblasts rescued the ciliary defects (Fig. 4c,d), as well as rapsyn’s localisation at centrosomes (Fig. 6a,b) and at the cilia base (Fig. 6c, Fig. 8), however, strongly supports the idea that rapsyn plays a role in ciliogenesis. This notion is further strengthened by our PLA assays revealing the association between rapsyn and γ-tubulin in PLA assays (Fig. 7a). Interestingly, MACF1 (microtubule-actin crosslinking factor) has been recently identified to bind to AChRs in a rapsyn-dependent manner^29^. MACF1 is a MT plus end-binding protein shown to promote persistent MT growth and to connect the actin and microtubule cytoskeletal network^77^. Moreover, MACF1 has been shown to be critical for cilia length and ciliogenesis in several cell types and in fibroblasts from patients with pathogenic variants in *MACF1*^78,79^. This novel interaction of rapsyn with MACF1 can be the link to its role in ciliogenesis. Interestingly, our PLA results also suggest a vicinity of rapsyn and NUP88 and that the rapsyn and NUP88 association is perturbed in FADS cells (Fig. 7a,b). The underlying molecular basis for this perturbation remains to be elucidated, but another yet to be identified linking protein may account for this effect.

Taken together, our data presented here strongly support the notion that impaired ciliogenesis is underlying FADS and that this contributes to the pleiotropic clinical features that coincide with the disease, in particular to craniofacial and skeletal defects. Moreover, PC are required for proper myoblast differentiation^80^, which suggests that also the muscular and movement defects seen in FADS individuals at least in part arise from impaired PC formation and growth.

## Material and methods

All experiments were carried out at room temperature, in triplicate and repeated at least three times unless otherwise stated. All experiments were carried out at matching passage numbers.

## Plasmids

The pcDNA3.1-rapsyn-FLAG construct was purchased from Genomics Online (ABIN4924584; Aachen, Germany). Rapsyn-Venus1 was generated by Gateway cloning using pDONR223-RAPSN (ABIN5316678; Genomics Online) as donor and pDEST-ORF-V1 (Addgene plasmid #73637, a gift from Darren Saunders) as destination vector. The presence and the integrity of the *RAPSN* insert was confirmed by sequencing.

Rapsyn-FLAG and rapsyn-Venus1 E162K mutants were generated by site-directed mutagenesis using the QuikChange Lightning site-directed mutagenesis kit (Agilent Technologies, CA, USA) following the manufacturer’s instructions. The primers were: forward 5′-CACACGCGGCACTTGAGCATGGCGTCA-3′, reverse 5′-TGACGCCATGCTCAAGTGCCGCGTGTG-3′. All constructs were verified by DNA sequencing.

### Cell culture and transfection

MRC5 (catalogue number AG05965-G), FADS (FADS 1; catalogue number GM11328), and HGPS (catalogue number AG 01972) fibroblasts were obtained from Coriell (Human Genetic Cell Repository; Coriell Institute, Hamden, NJ, USA). FADS 2 fibroblasts harbouring a homozygous c.484G > A (p.Glu162Lys) variant in the *RAPSN* gene (NM_005055.4) were from J. Breckpot, Catholic University Leuven, Belgium, as described previously^5^. MRC5 fibroblasts are from a 14 week aborted male Caucasian foetus, FADS 1 fibroblasts from a 20 week still born male Caucasian foetus, and FADS 2 fibroblasts from a 31 week old female foetus after caesarean section. FADS 2 fibroblasts are from a consanguineous family of Moroccan descend.

FADS 1 cells were grown in Minimum Essential Medium (MEM) Alpha (Lonza, Basel, Switzerland) supplemented with 15% foetal bovine serum (FBS) plus penicillin/streptomycin. HGPS cells were grown in MEM medium (Life Technologies Gibco, Gent, Belgium) supplemented with 15% FBS, non-essential amino acids plus penicillin/streptomycin. MRC5 and FADS 2 fibroblasts were grown in MEM medium supplemented with 10% FBS and penicillin/streptomycin. All cell lines were grown at 37 °C in 5% CO_2_ atmosphere. Cells were tested for mycoplasma contamination on a regular basis.

Transfection with siRNAs was carried out using Lipofectamine RNAiMAX (Life Technologies Invitrogen) following the instructions of the manufacturer. siRNAs were from Dharmacon (Lafayette, CO, USA): *RAPSN* (L-006550-00), *MUSK* (L-03158-00), *NUP88* (L-017547-01-0005), and non-targeting siRNAs (D-001810-10). Plasmids were transfected using Lipofectamine 3000 (Life Technologies Invitrogen) following the instructions of the manufacturer.

### EdU cell proliferation assay

For EdU incorporation, cells were plated on coverslips in a 24-well plate and grown to 70–80% confluency. Cells were next incubated for 1 h with medium containing 10 µM EdU, according to the manufacturer’s instructions (Click-iT Edu Alexa Fluor 594 Imaging Kit, Invitrogen). After incubation, cells were fixed for 15 min in 3.7% formaldehyde, washed twice in PBS containing 2% bovine serum albumin (BSA), permeabilised with PBS containing 0.5% Triton X-100 for 20 min, and washed twice in PBS/ 2% BSA. Cells were next incubated for 30 min with Click-iT reaction solution according to the manufacturer’s instructions, washed twice in PBS/2% BSA, washed two times in PBS. Coverslips were mounted with a drop of Mowiol-4088 (Sigma-Aldrich, St. Louis, MO, USA) containing DAPI (1 µg/ml; Sigma-Aldrich) and stored at 4 °C until viewed. Cells were imaged using a Zeiss Observer.Z1 microscope (Zeiss, Oberkochen, Germany). Images were recorded using the microscope system software and processed using Fiji/ImageJ. EdU positive cells were counted manually.

### Senescence assays

Cells were seeded in 6-well culture dishes at a density of 0.4 × 10^5^cells/well, 48 h prior to the experiment. Cellular senescence was analysed using the Senescence β-Galactosidase Staining Kit (Cell Signaling Technology, Cambridge, UK) according to the manufacturer's protocol. Development of the blue colour in senescent cells while the β-galactosidase was still on the culture dishes was evaluated using a Zeiss Observer.Z1 microscope. Images were recorded using the microscope system software and processed using Adobe Photoshop (Version 12.0; Adobe Systems, Mountain View, CA, USA). ß-galactosidase positive cells were counted manually.

### Cell cycle assays

Fibroblasts were plated in 10 cm dishes (0.6 × 10^6^ cells per dish), harvested after 24 h by trypsinization, collected with initial culture medium, washed with PBS, and fixed overnight at − 20 °C with 70% ethanol. For flow cytometric analysis, the cells were washed with PBS, incubated for 5 min with RNase A (0.2 mg/ml; Invitrogen), followed by a 15 min incubation at 37 °C with propidium iodide (PI; Sigma-Aldrich) solution (1 mg/ml PI, 10% Triton X-100 in PBS). Cells were analysed by flow cytometry using a FACS CantoII (BD Biosciences). Cell cycle distribution was analysed using the FlowJo software (BD Biosciences).

### Microtubule assays

In order to assess microtubule stability, cells were either put on ice for 10 min or treated with 10 µM (complete loss) or 170 nM (low polymerisation) of nocodazole (Sigma-Aldrich). For microtubule repolymerisation assays, cells were put on ice for 10 min and then incubated in a 37 °C humidified 5% CO_2_ atmosphere for 0, 30 and 90 s. Cells were imaged using a Zeiss Observer.Z1 microscope. Images were recorded using the microscope system software and processed using Fiji/ImageJ and Adobe Photoshop. Microtubule nucleation was counted manually.

### Doubling time

Cells were seeded in 6-well culture plates at a density of 0.4 × 10^5^ cells/well. Cells were counted using a TC20 Automated Cell Counter (Bio-Rad Laboratories, California, USA) at indicated time points, over a range of at least 168 h. Only the linear parts of the count data were used in the analysis.

Growth curve analysis (GCA), growth constant k (slope of linear regression line), and doubling time T_d_ were calculated using the multilevel regression technique using the open-source R Studio (version 3.6.0) and R Studio (version 1.2.1335; https://www.R-project.org) as described earlier^81^. By using GCA, both group-level and individual-level effects on cellular growth were analysed. The linear model (m.1; Fig. 2a) suggests effects of the different cell lines on proliferation and the intercept model (m.0; Supplemental Fig. S7b) suggests constant differences in proliferation randomly assigned to the different cell lines. Statistical comparison (Pearson's χ^2^ test with two degrees of freedom of the models) revealed that the linear regression model m.1 was used as regression model of choice (*p* < 0.001).

### Confocal imaging

Cells were grown on coverslips in 24-well plates and fixed either with 2% formaldehyde for 15 min or -20 °C methanol (for γ-tubulin and centrin-3 staining) for 10 min, washed three times for 10 min with PBS, and permeabilized with PBS/1% BSA/ 0.2% Triton X-100 for 10 min on ice. Next, the cells were washed three times for 10 min in PBS/2% BSA and incubated with the appropriate primary antibodies for 1 h or over-night at 4 °C, washed three times in PBS /2% BSA and incubated with the appropriate secondary antibodies for 1 h, washed four times 10 min with PBS and mounted with a drop of Mowiol-488 containing DAPI (1 µg/ml). Cells were imaged using a Zeiss LSM-710 confocal laser-scanning microscope in AiryScan super-resolution mode (Zeiss, Oberkochen, Germany). Images were recorded and processed using the microscope system software and further edited using Fiji/ImageJ and Adobe Photoshop.

The following antibodies were used as primary antibodies: monoclonal mouse anti-lamin A/C (1:30; ab40567; Abcam, Cambridge, UK), mouse anti-γ-tubulin (1:1000; ab11316; Abcam), mouse anti-ß-tubulin (1:1000; clone KMX-1; Merck Millipore, Massachusetts, USA), mouse anti-acetylated α-tubulin (1:1000; T7451; Sigma-Aldrich), mouse anti-DOK7 (1:50; NBP2-02073; Novus Biologicals, Abingdon, UK), mouse anti-NUP88 (1:500; 611896; BD Biosciences, San Jose, CA, USA), mouse anti-Ki67 (1:100; NCL-L-Ki67-MM1; Novocastra Laboratories, Newcastle upon Tyne, UK), and rat anti-α-tubulin (1:1000; MCA78G; Bio-Rad AbD Serotec). Polyclonal rabbit lamin B1 (1:300; ab16048; Abcam), rabbit anti-γ-tubulin (1:100; 15176-1-AP; Proteintech, Mancester, UK), rabbit anti-centrin-3 (1:1000; PA5-35,865; Thermo Scientific, Rockford, USA), rabbit anti-acetylated α-tubulin (1:800; 5335; Cell Signaling, Leiden, The Netherlands), rabbit anti-Arl13b (1:200; 17,711-1-AP; Proteintech), rabbit anti-rapsyn (1:200; NBP1-85,537; Novus Biologicals), and rabbit anti-MuSK (1:100; PA5-14,705; Thermo Scientific).

Secondary antibodies were the corresponding goat anti-mouse IgG Alexa Fluor 568, goat anti-rabbit IgG Alexa Fluor 568, goat anti-mouse IgG Alexa Fluor 488, goat anti-rabbit IgG Alexa Fluor 488, and goat anti-human IgG Alexa Fluor 568. All secondary antibodies were diluted 1:1000 and purchased from Invitrogen.

### Western blotting

Cells were lysed in lysis buffer (50 mM Tris–HCl, pH 7.8, 150 mM NaCl, 1% Nonidet-P40 and protease inhibitor cocktail tablets (Roche, Basel, Switzerland)). 30 µg of protein were loaded and separated by sodium dodecyl sulphate–polyacrylamide gel electrophoresis (SDS-PAGE). The proteins were transferred onto a PVDF membrane (Immobilon-P, Merck Millipore) and the membranes were blocked with TBS containing 0.1% Tween 20 and 5% non-fat dry milk for 1 h. The membranes were then incubated over-night at 4 °C in blocking solution containing a primary antibody followed by washing 3 × in TBS/ 0.1% Tween 20/ 5% non-fat dry. The membranes were next incubated with secondary antibodies for 1 h, washed 3 × in TBS/0.1% Tween 20 and developed. X-ray films were scanned and processed using Fiji/ImageJ. Gel densitometry measurement was done with Fiji/ImageJ.

The following antibodies were used as primary antibodies: monoclonal mouse anti-lamin A/C (1:200; ab8984, Abcam), mouse anti-NUP88 (1:1000), mouse anti-DOK7 (1:1000), mouse anti-CRM1 (1:1000; 611832, BD Biosciences), and mouse anti-ß-tubulin (1:1000). Polyclonal rabbit anti-lamin B1 (1:300), rabbit anti-rapsyn (1:500), rabbit anti-MuSK (1:1000), rabbit anti-actin (1:1000; A2066, Sigma-Aldrich), rabbit anti-acetylated α-tubulin (1:1000), rabbit anti-detyrosinated α -tubulin (1:1000), and rabbit anti-α-tubulin (1:4000; ab18251, Abcam).

Secondary antibodies were either alkaline phosphatase-coupled IgG antibodies (1:10.000; Sigma/Aldrich) or horseradish peroxidase-coupled IgG antibodies (1:8000; Cell Signaling Technology).

### Proximity ligation assay

All antibodies used for proximity ligation assay (PLA) were diluted in blocking solution. Antibodies rabbit anti-rapsyn, rabbit anti-γ-tubulin, mouse anti-γ-tubulin, and mouse anti-NUP88 were incubated at 4 °C over-night in a humidified chamber. Excess antibodies were removed by three washing steps using 0.1% Triton X-100 in PBS for 5 min. PLA was performed using Duolink PLA Fluorescent Detection (red) with anti-mouse PLUS and anti-rabbit MINUS oligonucleotides (Sigma-Aldrich). PLA was performed as described elsewhere^82^. Cover slips were mounted onto microscope slides with Mowiol-488 containing DAPI. Cells were imaged using a Zeiss Observer.Z1 microscope. Images were recorded using the microscope system software and processed using Fiji/ImageJ and Adobe Photoshop. PLA foci were counted using a macro written on Fiji/ImageJ.

### Statistical analyses

All plots and statistics (despite GCA models) were generated using GraphPad Prism (Version 8;GraphPad Software Inc., CA, USA) or Apple Numbers (Apple Inc., CA, USA). Two-tailed t-test was performed. During evaluation of the results a confidence interval α of 95% and *p* values lower than 0.05 were considered as statistically significant.

### Image design

Schematic representations were designed using the open-source software Inkscape 0.91 (by running XQuartz 2.7.11 on macOS). Colours were adapted from https://www.ColorBrewer.org by Cynthia A. Brewer.

## Supplementary information

Supplementary Information
