# Supplementary material for: Centrosome and ciliary abnormalities in fetal akinesia deformation sequence human fibroblasts
Source: Sci Rep. 2020 Nov 9;10:19301. doi: 10.1038/s41598-020-76192-1 (PMC7652866; doi:10.1038/s41598-020-76192-1)
Supplement: Supplementary file 1 — Supplementary Information [file 41598_2020_76192_MOESM1_ESM.pdf]

Centrosome and ciliary abnormalities in fetal akinesia deformation sequence human fibroblasts

Ramona Jühlen, Valérie Martinelli, Chiara Vinci, Jeroen Breckpot, and Birthe Fahrenkrog

Supplementary figures

Supplementary figures

Figure S1: Mitotic FADS cells exhibit abnormal sloppy microtubule spindles, but spindle stability is not affected upon nocodazole treatment. (**a**) MRC5 and FADS 1 fibroblasts were cold-treated and mitotic cells stained for β-tubulin (green) and DAPI (blue). (**b**) MRC5 and FADS 1 fibroblasts were treated with high (10 μM, complete loss of polymerisation) or low (170 nM, little polymerisation) concentrations of nocodazole and mitotic cells stained for β-tubulin (green) and DAPI (blue). Scale bars, 10 μm.

Figure S2: FADS affects cilia growth, but not cilia resorption. (**a**) Representative immunofluorescence images of anti-acetylated α-tubulin (ac-tubulin; magenta) and anti-detyrosinated α-tubulin (detyr-tubulin; magenta) staining in serum-starved MRC5 and FADS fibroblasts grown on cross-bow-shaped micropattern. Actin (green) was visualised by phalloidin-Alexa Fluor 488 and DNA by DAPI (blue). (**b**) Cilia resorption after release from 48 h serum starvation was comparable in MRC5 and FADS fibroblasts. PC, primary cilia. (**c**) Laminopathies do not coincide with ciliary defects. Atyp progeria, atypical progeria; EDMD, Emery Dreifuss muscular dystrophy; FPLD, Familial partial lipodystrophy; HGPS, Hutchison Gilford progeria syndrome. (**d**) Representative immunofluorescence images of anti-acetylated α-tubulin (ac-tub; green) and anti-detyrosinated α-tubulin (detyr-tub; green) stainings in growing MRC5 and FADS fibroblasts. Detyrosinated α-tubulin stainings show aggregations in FADS cells. Scale bars, 10 μm.

Figure S3: (**a**) MuSK-depleted MRC5 fibroblasts exhibit shorter PCs. MRC5 cells were transfected with a siRNA against MuSK and cells were serum starved for 48 h, fixed, and stained for acetylated α-tubulin (ac-tubulin; green) and Arl13b (green). DNA (blue) was visualised by DAPI staining. Scale bars, 5 μm. (**b**) MRC5 cells were transfected with the indicated siRNAs and the protein levels of rapsyn and NUP88 were determined by Western blot analysis and (**c-d**) densitometric quantification using Fiji/ImageJ. Data present the mean ±SD of at least three independent experiments. Full-length blots are shown in Fig. S11. $^{**}p<0.01$, ns p not significant, t-test, two-tailed.

Figure S4: Co-localization of nucleoporins in MRC5 cells with centrosomes. (**a**) MRC5 cells were immunostained with anti-NUP214, anti-NUP62, anti-NUP93, and anti-NUP153 antibodies, respectively, (red) and anti-γ-tubulin antibodies (green). White arrows indicate the respective position of the centrosome. Validation of the specificity of (**b**) the rapsyn and (**c**) the NUP88 antibodies and their respective localisation of at centrosomes. MRC5 cells were treated for 72 h with the indicated siRNAs. Centrosomes were visualised by anti-γ-tubulin antibodies. Shown are representative confocal images. Scale bars, 10 μm.

Figure S5: Validation of the specificity of the rapsyn and the NUP88 antibodies in MRC5 and HeLa cells. (**a**-**b**) Cells were immunostained with anti-NUP88 (green). Mean grey values at the nuclear rim were quantified automatically with Fiji/ImageJ and normalised. (**c**-**d**) Cells were immunostained with anti-rapsyn (green). Mean grey values at the centrosome were quantified automatically with Fiji/ImageJ and normalised. Cells were treated for 72 h with the indicated siRNAs. Shown are

representative confocal images. DNA (blue) was visualised by DAPI staining. Scale bars, 10 μm.

Figure S6: (**a**) DOK7 (magenta) localises to the axoneme of the PC in sub-confluent MRC5, FADS 1, and FADS 2 fibroblasts that were serum-starved for 48 h. Cilia were visualised by anti-acetylated α-tubulin staining (ac-tub; green). (**b**) No cilia association was seen for MuSK (magenta). DNA was visualised by DAPI (blue). White arrows indicate the respective position of the PC. Shown are representative confocal images. Scale bars, 10 μm.

Figure S7: (**a**) Proximity ligation assay (PLA) control experiments. MRC5 cells were processed for PLA using either the probes only or the antibodies only in order to rule out false positive PLA foci (red). Scale bars, 10 μm. (**b**) Intercept regression model of Figure 2 A. Growth curve analysis was done using multilevel regression technique using R. The intercept model presented here suggests constant differences in proliferation randomly assigned to the different cell lines and the linear model (Fig. 2a) suggests effects of the different cell lines on proliferation. Statistical comparison of these two models revealed that the linear model is our regression model of choice. Data points show the mean at the specific time point and the point range show the SEM.

Figure S8: Full-length blots of blots shown in Fig. 1d.

Figure S9: Full-length blots of blots shown in Fig. 4e.

Figure S10: Full-length blots of blots shown in Fig. 4g.

Figure S11: Full-length blots of blots shown in Fig. S3b.

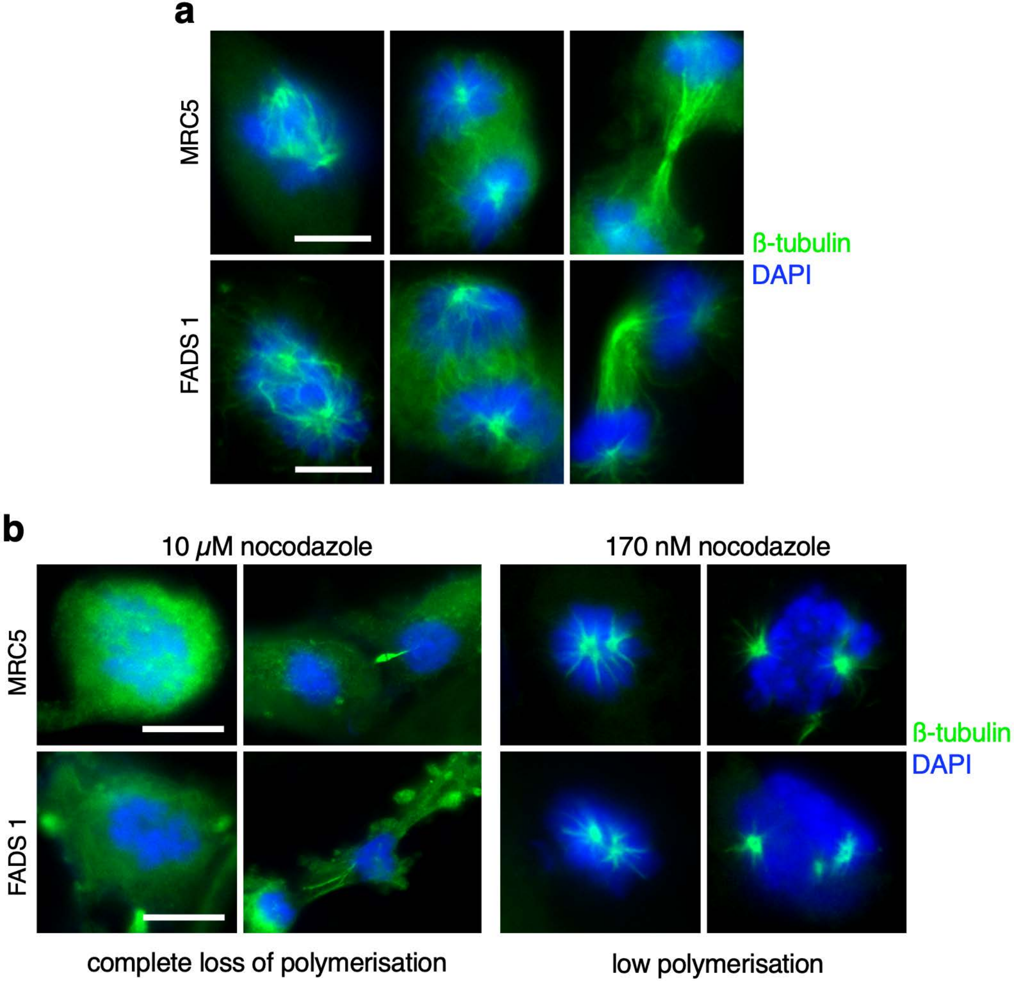
a
MRC5
FADS 1
ß-tubulin
DAPI
b
10 μM nocodazole
170 nM nocodazole
MRC5
FADS 1
ß-tubulin
DAPI
complete loss of polymerisation
low polymerisation

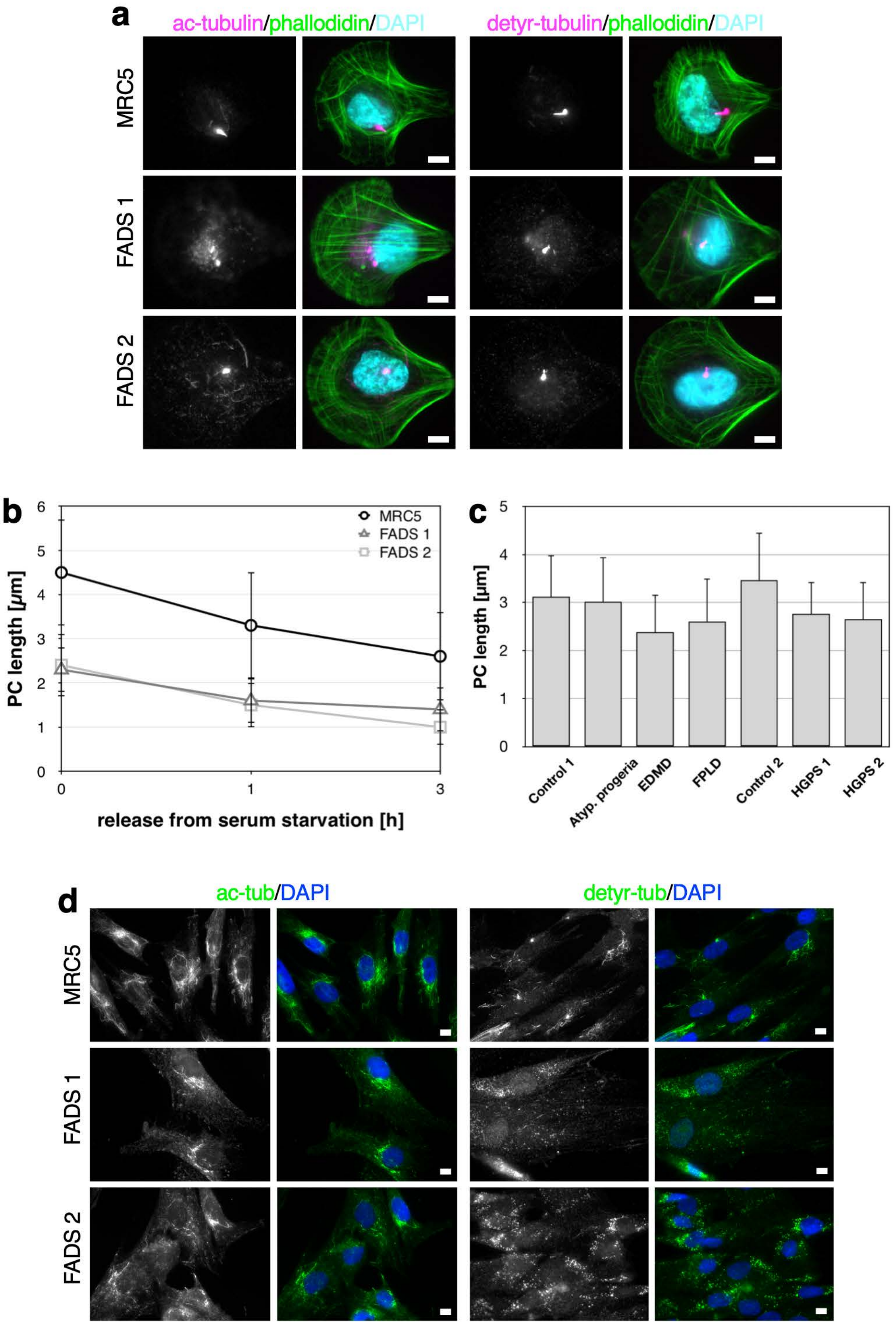
a
ac-tubulin/phallodidin/DAPI
detyr-tubulin/phallodidin/DAPI
MRC5
FADS 1
FADS 2
b
MRC5
FADS 1
FADS 2
PC length [μm]
release from serum starvation [h]
0
1
3
c
PC length [μm]
Control 1
Atyp. progeria
EDMD
FPLD
Control 2
HGPS 1
HGPS 2
d
ac-tub/DAPI
detyr-tub/DAPI
MRC5
FADS 1
FADS 2

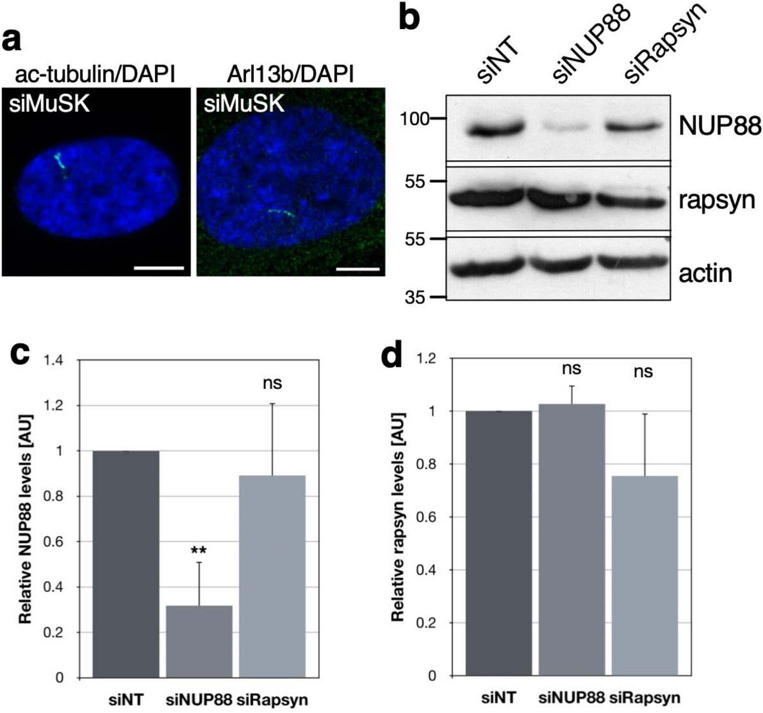
a
ac-tubulin/DAPI
Arl13b/DAPI
siMuSK
siMuSK
b
siNT
siNUP88
siRapsyn
100
55
55
35
NUP88
rapsyn
actin
c
Relative NUP88 levels [AU]
1.4
1.2
1
0.8
0.6
0.4
0.2
0
**
ns
siNT
siNUP88
siRapsyn
d
Relative rapsyn levels [AU]
1.2
1
0.8
0.6
0.4
0.2
0
ns
ns
siNT
siNUP88
siRapsyn

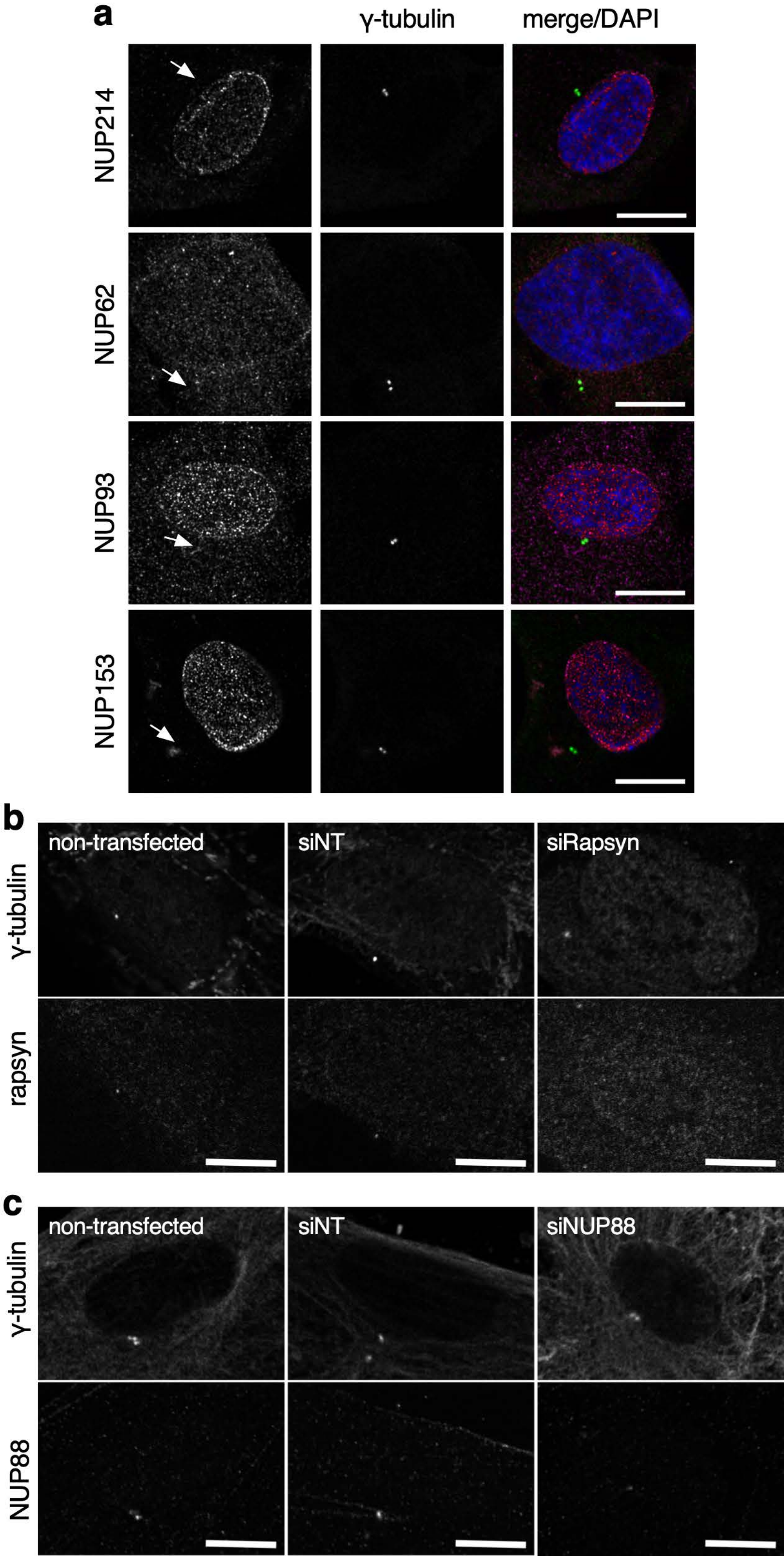

a
γ-tubulin
merge/DAPI
NUP214
NUP62
NUP93
NUP153
b
non-transfected
siNT
siRapsyn
γ-tubulin
rapsyn
c
non-transfected
siNT
siNUP88
γ-tubulin
NUP88

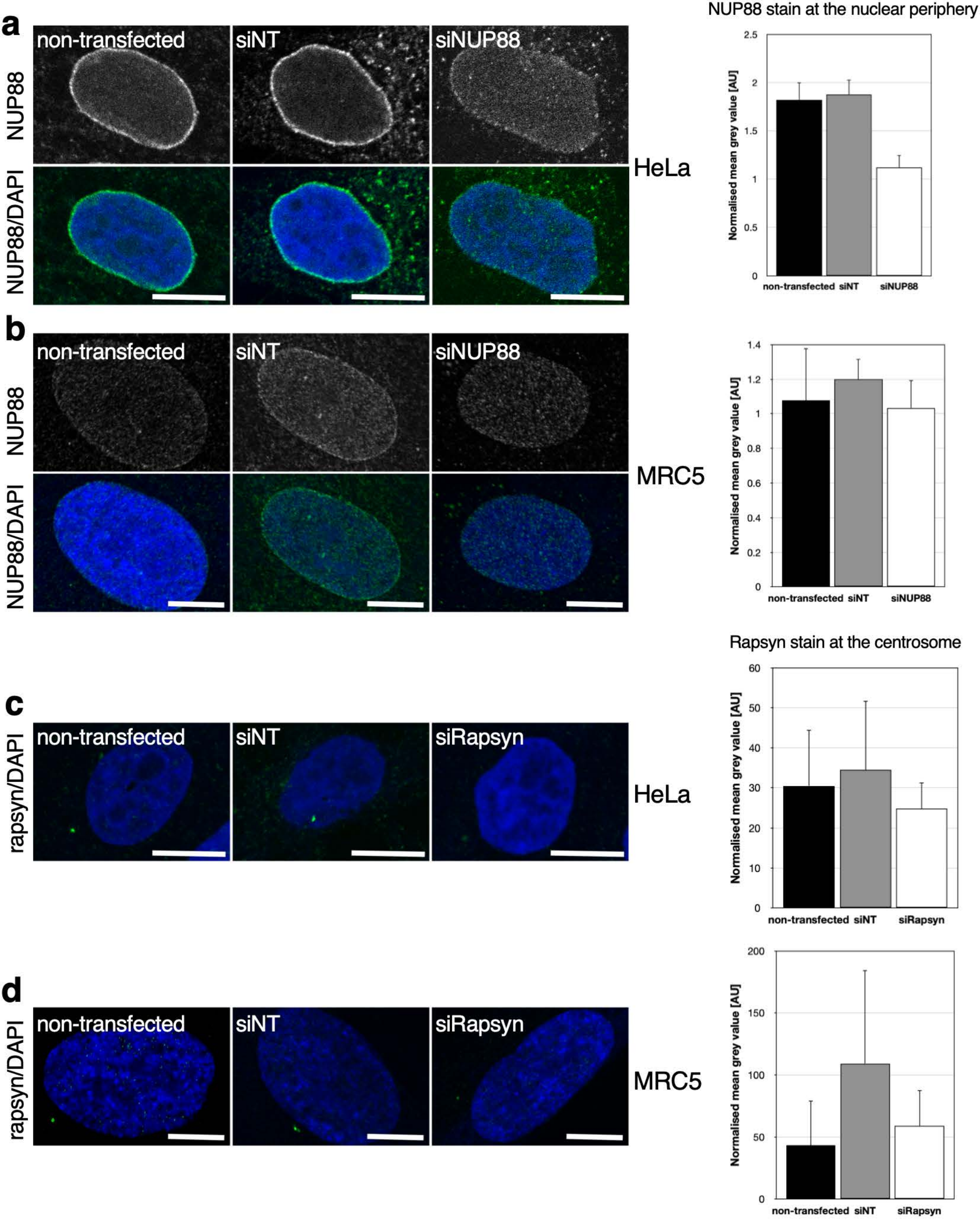

a
non-transfected
siNT
siNUP88
NUP88
NUP88/DAPI
HeLa
NUP88 stain at the nuclear periphery
Normalised mean grey value [AU]
non-transfected
siNT
siNUP88
b
MRC5
c
rapsyn/DAPI
siRapsyn
Rapsyn stain at the centrosome
d

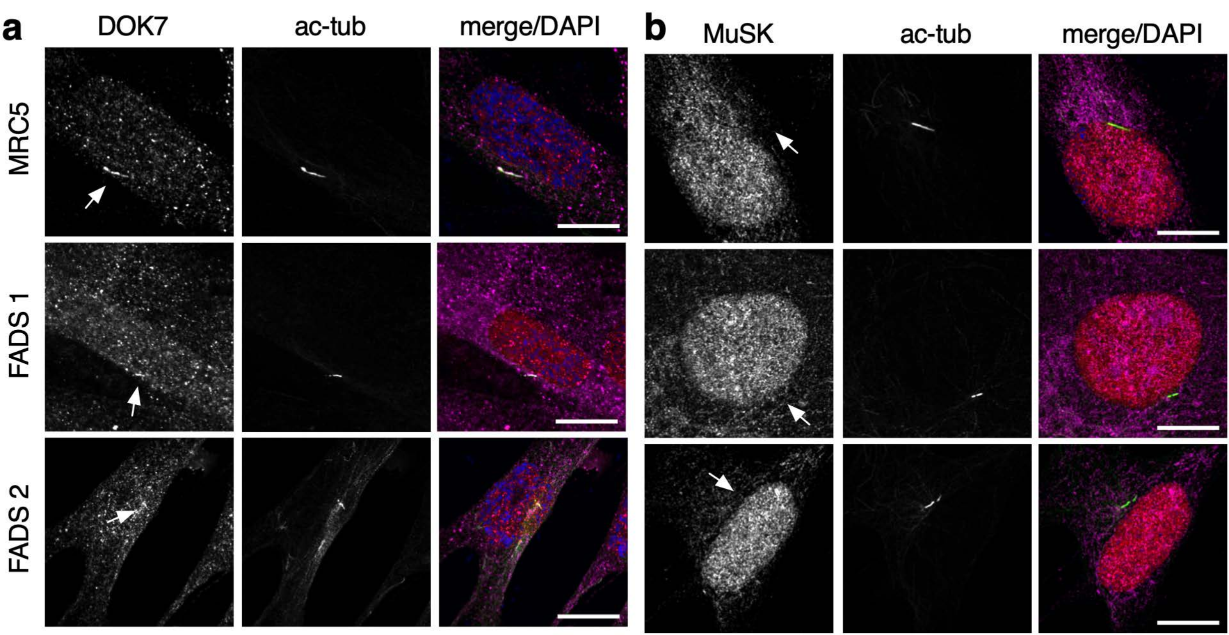
a
DOK7
ac-tub
merge/DAPI
b
MuSK
ac-tub
merge/DAPI
MRC5
FADS 1
FADS 2

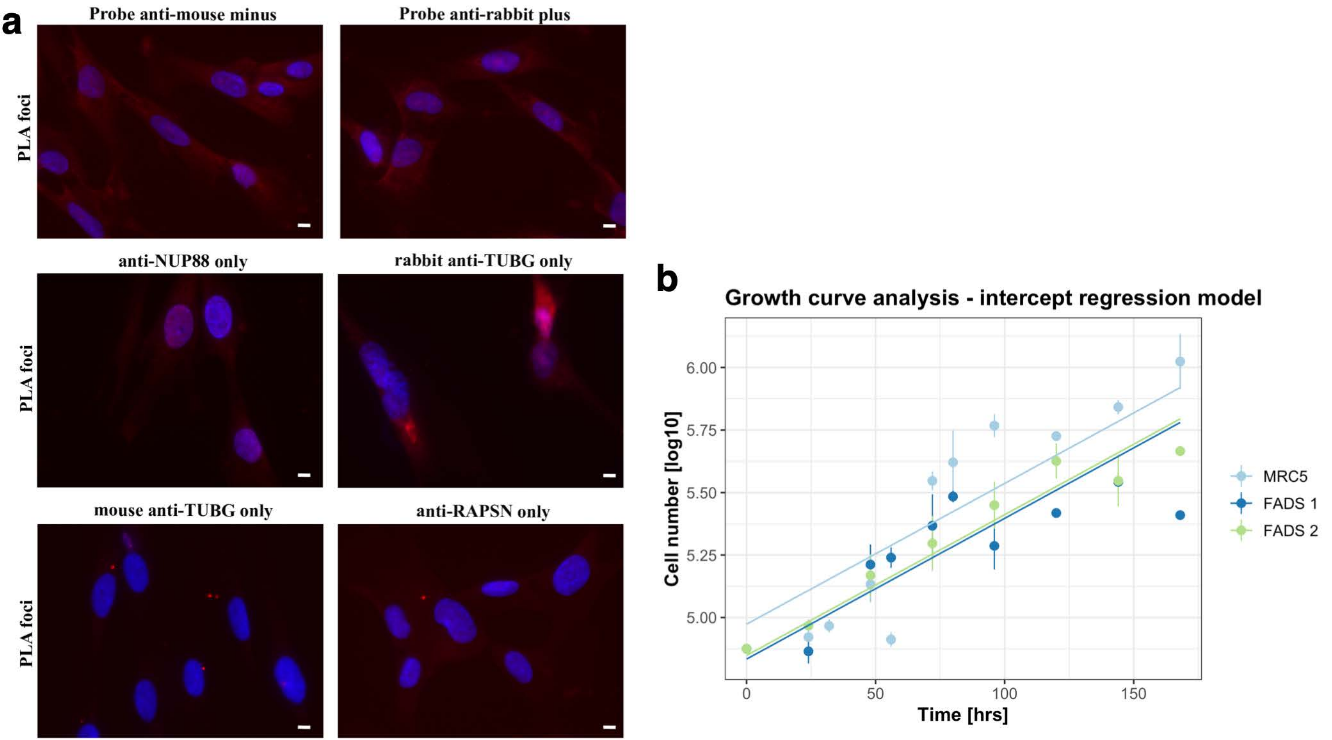
a
Probe anti-mouse minus
Probe anti-rabbit plus
PLA foci
anti-NUP88 only
rabbit anti-TUBG only
PLA foci
mouse anti-TUBG only
anti-RAPSN only
PLA foci
b
Growth curve analysis - intercept regression model
Cell number [log10]
6.00
5.75
5.50
5.25
5.00
0
50
100
150
Time [hrs]
MRC5
FADS 1
FADS 2

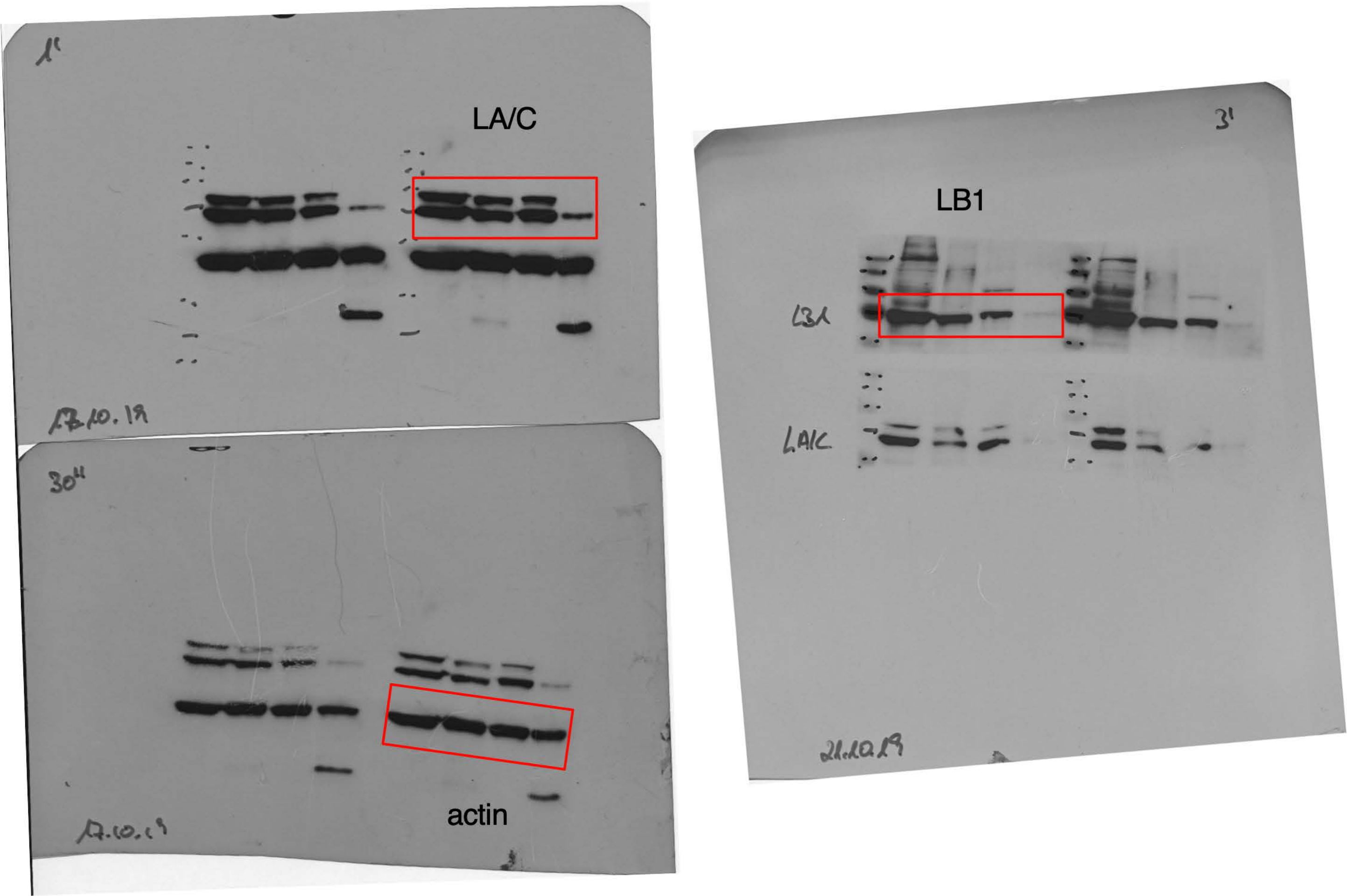
1'
LA/C
17.10.19
30''
actin
17.10.19
3'
LB1
LB1
LA/C
21.10.19

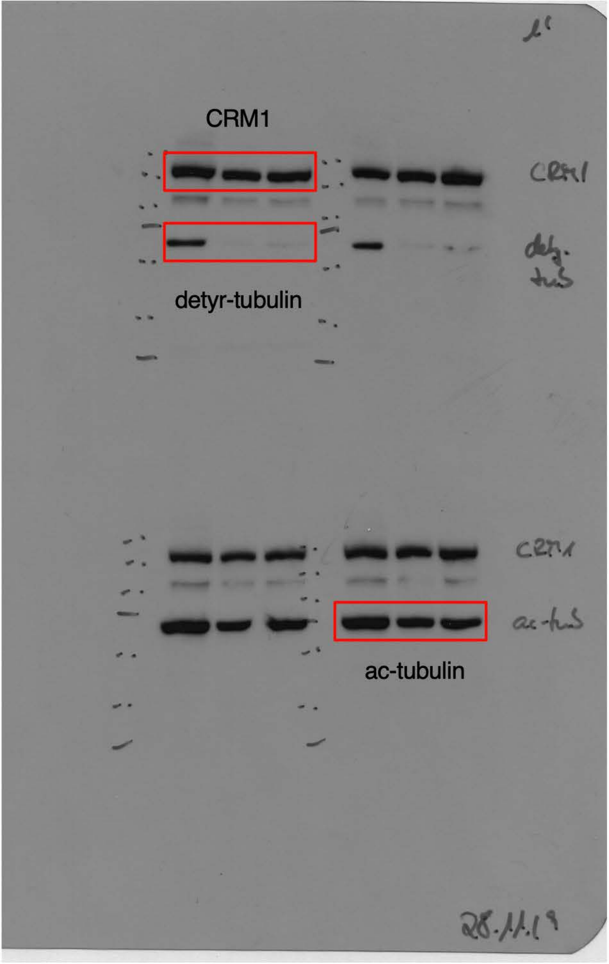
CRM1
detyr-tubulin
ac-tubulin
28.11.19

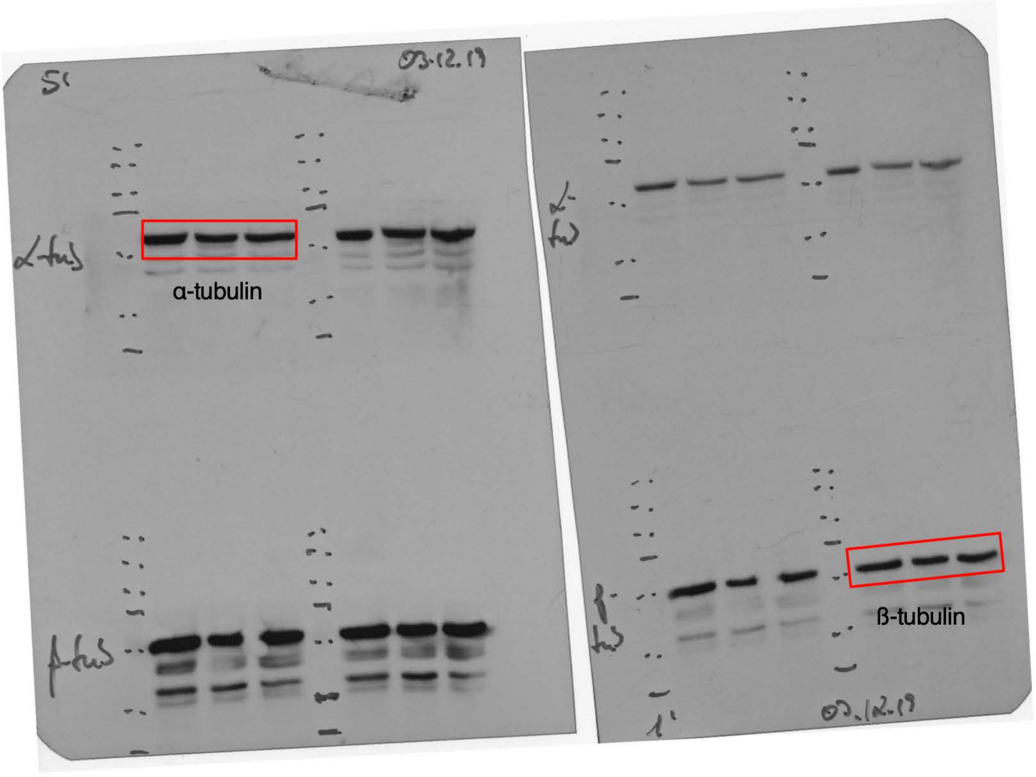
03.12.19
α-tubulin
ß-tubulin
03.12.19

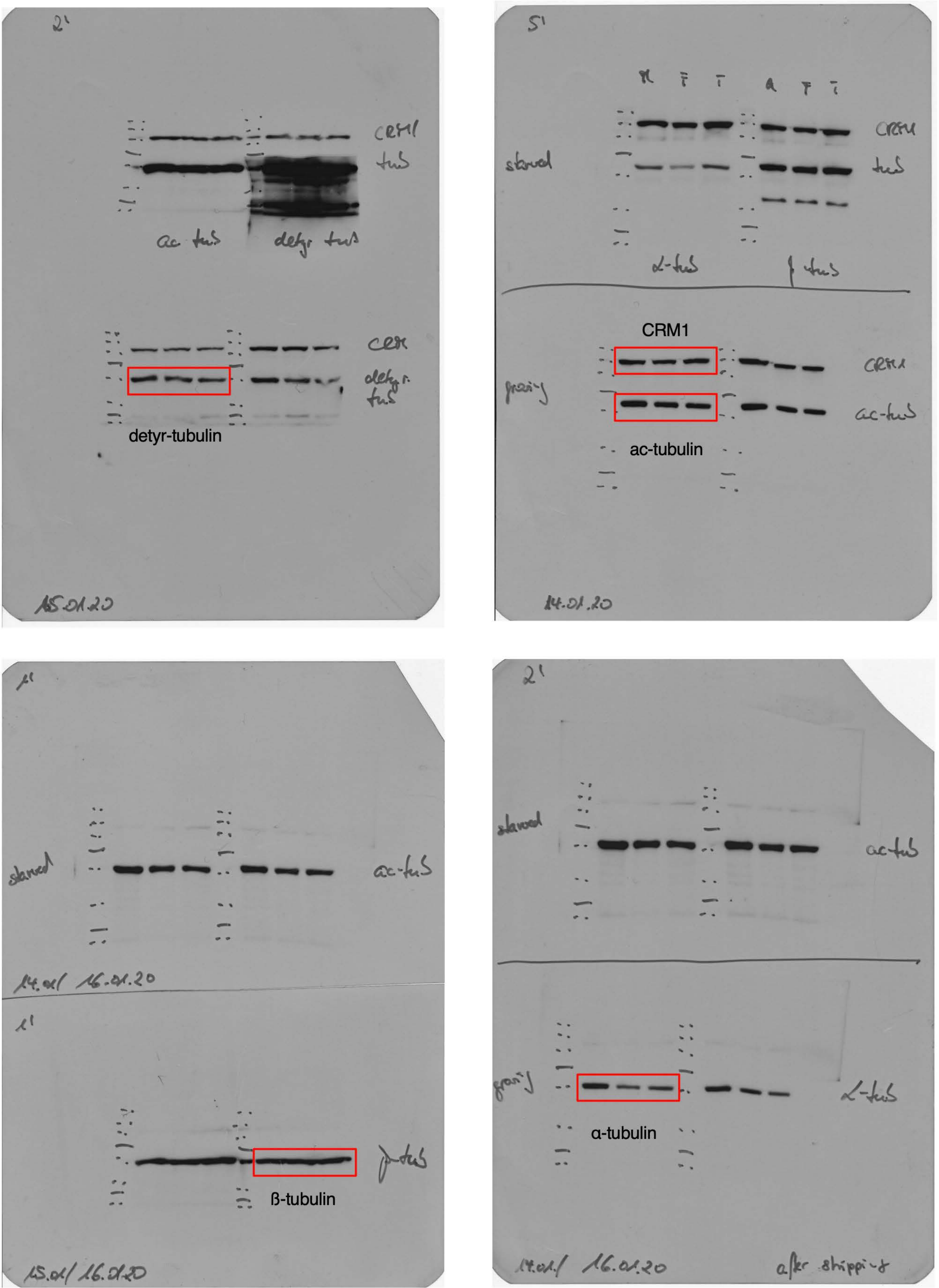

CRM1
ac-tubulin
detyr-tubulin
ß-tubulin
α-tubulin
starved
15.01.20
14.01.20
14.01/16.01.20
15.01/16.01.20
after shipping

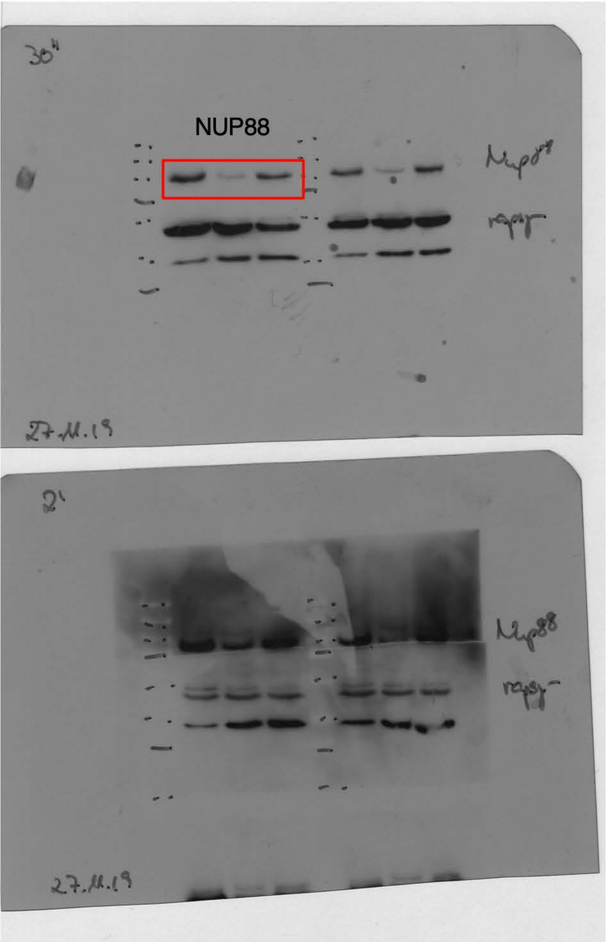
30''
NUP88
27.11.19
2'
Nup88
27.11.19

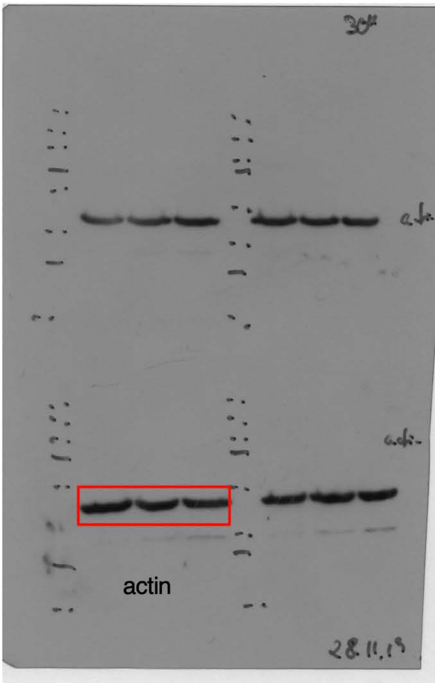
30''
actin
28.11.19

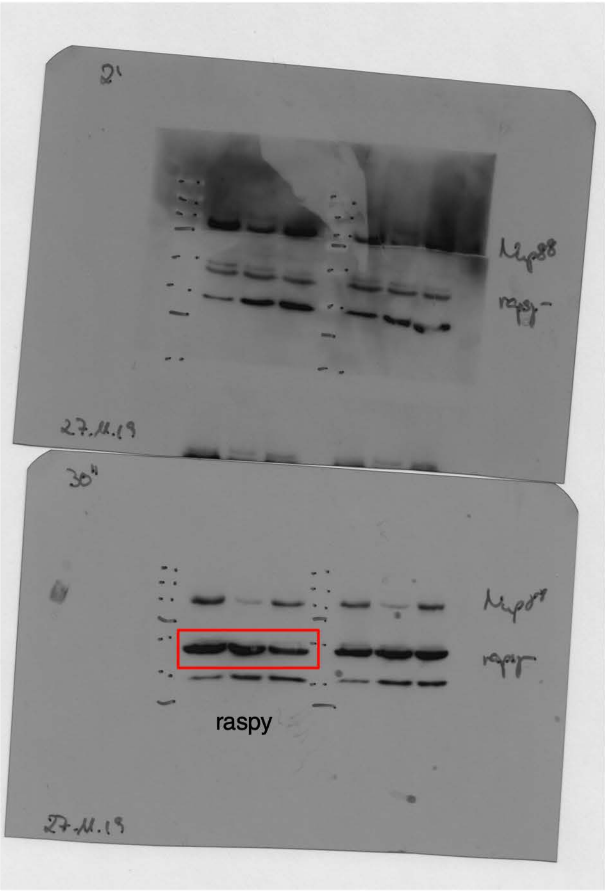
2'
Nup88
27.11.19
30''
raspy
27.11.19

Table S1: Cilia localisation of rapsyn and NUP88 in control and FADS fibroblasts.

	**MRC5**	**FADS 1**	**FADS 2**
rapsyn	97.4 ± 1.2	94.8 ± 2.6	97.0 ± 0.9
NUP88	94.4 ± 2.9	86.9 ± 10.6	95.2 ± 1.1

Numbers are in percent ± standard deviation. Absolute number of analysed cilia: rapsyn: MRC5, 233; FADS 1, 219; FADS 2, 243; NUP88: MRC5, 200; FADS 1, 289; FADS 2, 146
